# Explaining trunk strength variation and improvement following resistance training in people with chronic low back pain: clinical and performance-based outcomes analysis

**DOI:** 10.1038/s41598-025-93280-2

**Published:** 2025-03-13

**Authors:** Shouq Althobaiti, David Jiménez‑Grande, Janet A. Deane, Deborah Falla

**Affiliations:** 1 Centre of Precision Rehabilitation for Spinal Pain (CPR Spine), School of Sport, Exercise and Rehabilitation Sciences, College of Life and Environmental Sciences, University of Birmingham, Birmingham, UK; 2 Physical Therapy Department, College of Applied Medical Science, Taif University, Taif, Saudi Arabia

**Keywords:** Low back pain, Trunk muscle strength, Resistance training, Patient-reported outcomes, Performance-based outcomes, Rheumatology, Musculoskeletal system

## Abstract

A multitude of variables contribute to the variation of trunk strength in individuals with chronic low back pain (CLBP). This study investigated a range of variables to determine which variables contribute most to variation in trunk isometric strength and gains in strength following resistance training in people with CLBP. Outcome measures were recorded from 20 participants with CLBP both at baseline and following resistance training. Regression analyses were applied with the average trunk maximum voluntary isometric torque as the dependent variable. Variance in baseline trunk flexion strength (*R*^2^ = .66) was explained by demographic covariates and a measure of trunk muscle co-activation. The baseline trunk extension strength variance (*R*^2^ = .65) was explained by demographic covariates and lumbar erector spinae (LES) activity during a maximum trunk extension contraction. Demographic variables, trunk muscle co-activation, baseline trunk flexion strength, level of physical function, and pain intensity over the past week influenced the change in trunk flexion strength after training (*R*^2^ = .93). Demographic variables and LES muscle activity explained the variance in trunk extension strength at follow-up (*R*^2^ = .64). This study supports the major influence of sex, physical function and baseline strength and muscle activity, on the variation in maximum trunk strength in participants with CLBP at baseline and gains in trunk muscle strength following progressive resistance training.

## Introduction

Chronic low back pain (CLBP) is a prevalent and debilitating condition that affects 577 million individuals, approximately 7.5% of the global population, significantly impacting their quality of life^1,2^. CLBP is a multifactorial phenomenon influenced by a myriad of interconnected factors such as biophysical, comorbidities, genetic, social and psychological factors^3^. Significant biophysical contributing factors include the ability of the trunk muscle to produce force and sustain that force production (strength and endurance), which play a crucial role in the control of spinal motion and the maintenance of spinal health^4^.

Decreased isometric trunk muscle strength is frequently observed in individuals with CLBP compared to healthy individuals^5,6^, which is associated with LBP severity^7^. However, some studies have reported contrasting evidence where no difference in trunk muscle strength was reported between individuals with CLBP and healthy controls^8,9^. This discrepancy may be partly attributed to the heterogeneity of LBP populations and differences in measurement protocols, such as variations in participant positioning and contraction type used when measuring strength (isometric vs isokinetic)^10^.

The relation between physical fitness measures, including strength, and other demographic and anthropometric variables such as age, sex, and weight, are recognised^11,12^. For example, being female has been linked to reduced trunk flexion strength, and both female sex and lower body mass index (BMI) are associated with diminished trunk extension strength^13^. In a related study, 68 participants were divided into three age-matched groups (normal weight, overweight, and obese) to investigate the influence of obesity on maximal trunk extension strength^14^. While absolute trunk extension strength was similar across groups, obesity was associated with significantly lower strength when normalised to body mass and fat-free mass (FFM). In this study, FFM and age explained 60% of the variability in trunk strength^14^. Similarly, age is strongly linked to muscle strength reduction. Yassierli et al.^15^ found that older individuals (mean age 60.8 ± 4.0 years) had approximately 27% lower trunk extension maximal voluntary contraction (MVC) compared to a younger group (mean age 21.5 ± 1.2 years). In CLBP individuals, multivariate analysis showed that females and older participants had significantly less extensor strength, with females also exhibiting lower flexion strength. Interestingly, symptom duration (acute vs chronic LBP) and lifestyle factors (sedentary, semi-active, active) did not significantly affect strength in this population^13^.

In addition to demographic characteristics, disability, pain intensity, pain anticipation, and pain avoidance behaviours have been frequently suggested to be associated with variation in trunk strength in individuals with CLBP^6,16–18^. The relation between these variables could also contribute to sub-optimal performance during strength tests. The extent to which patients perceive their pain as disabling directly impacts their level of physical activity^19,20^. Furthermore, correlations have been found between fear of movement, higher pain levels, and greater disability^21^. Due to pain-related fear, tasks requiring maximal effort, such as strength testing, are often avoided, leading to submaximal effort and poorer outcomes^22,23^. However, a recent study showed that neither pain, disability nor kinesiophobia are among the variables that explain variation in isometric trunk flexion and extension strength in individuals with CLBP^24^. Psychological factors, such as fear-avoidance behaviours and pain-related fear, are associated with alterations in spinal motor behaviour, which refers to changes in spinal motion characteristics, including reduced amplitude and velocity of spinal movement, as well as increased trunk muscle activity^25,26^.

Additionally, variability exists between individuals in their responsiveness to the exercise intervention, with some experiencing greater improvements in strength than others^27^. The variability of individual exercise responsiveness could indicate true differences in individual responses or simple random variations within participants. The effect of an intervention on strength gain could also be influenced by changes in other outcome variables. Keller et al.^28^ reported that changes in pain, fear-avoidance beliefs, and self-efficacy for pain and treatment accounted for 46% of the variation in muscle strength at 1 year following either lumbar fusion or cognitive intervention and exercises. Whilst most studies have focused on the role of patient-reported outcomes and their influence on trunk strength, potential associations between different functional parameters such as muscle endurance, and muscle activation/co-activation remain unexplored. Thus, it is unclear whether functional parameters may explain variation and/or the extent of gains in trunk muscle strength in individuals with CLBP^29^. There is no study to date that has comprehensively examined both patient-reported outcomes (e.g., pain, disability, fear of movement) and performance-based outcomes (e.g., trunk strength) to understand how these variables collectively influence trunk strength variability and gain in individuals with CLBP. This research gap highlights the need for further investigation to better understand the interplay between subjective and objective factors contributing to trunk strength variation in this population.

Therefore, this study aimed to conduct a comprehensive evaluation of both patient-reported outcome measures (PROMS) and performance-based outcome measures (PerFOMs) and to determine whether any of these variables are associated with variation in maximum isometric trunk strength in participants with CLBP and gains in trunk muscle strength following a progressive resistance training intervention. Isometric testing was chosen due to its safety and reproducibility, allowing for precise strength measurements without movement variability^30^, and its relevance in assessing core stability, which is crucial for injury prevention and performance^31–33^.

## Results

### Participant characteristics and outcome data

In total, 20 participants (13 female) with CLBP, with a mean age of 33.2 (7.3) years were included. An overview of participants’ characteristics and both dependent and independent variables are shown in Table 1. The Intraclass Correlation Coefficient (ICC) for the consistency of MVIC performance across sessions indicates moderate to excellent reliability in the strength measurements^34^. The detailed ICC values and additional reliability analyses are provided in (Supplementary File S1). After identifying and replacing one outlier from the mean trunk flexion strength with the closest value^35^, all assumptions for regression analysis were met.

**Table 1:** Participants (n = 20) characteristics, dependent, and independent variables.

	Mean (SD)
Independent variables	
Sex, Females (%)	13 (65%)
Age (years)	33.20 (7.30)
Weight (kg)	70.80 (12.72)
Flex contraction pain (NPRS)	2.98 (1.37)
Ext contraction pain (NPRS)	3.23 (1.04)
Pain past week (NPRS)	4.55 (2.16)
Pain 24 h (NPRS)	3.85 (1.87)
RA mean RMS (µV) during flexion MVIC	14.49 (10.39)
LES mean RMS (µV) during extension MVIC	15.26 (9.81)
LES-RA Co-activation (Flex CI %)	180.59 (163.07)
RA-LES Co-activation (Ext CI %)	20.41 (8.42)
Endurance time (sec)	228.90 (86.14)
Average LES mean frequency (MNF)	85.86 (11.97)
Disability (ODI%)	41.10 (9.09)
Kinesiophobia (TSK)	38.30 (8.54)
Average physical functioning (SF36) (0–100)	62.75 (16.97)
Role limitations due to physical health (SF36) (0–100)	53.75 (43.13)
Dependent variables	
Trunk flexion-baseline (N/m)	84.73 (39.40)
Trunk extension-baseline (N/m)	99.55 (59.05)
Trunk flexion-follow up (N/m)	99.96 (33.60)
Trunk extension-follow up (N/m)	108.84 (41.12)

Results are expressed as mean, (standard deviation). Kg, Kilogram; NPRS, numeric pain rating scale; RA, rectus abdominus; LES, lumber erector spinae; RMS, root mean square; CI, co-activation index; sec, seconds; MNF, mean spectral power frequency; ODI, Oswestry disability index; TSK, Tampa Scale for Kinesiophobia; SF36, Short Form-36; N/m, Newton/ meter; MVIC, maximum voluntary isometric contraction.

### Correlations of dependent and independent variables

An overview of all correlations is presented in Table 2. Normality was checked using Shapiro–Wilk test and all dependent variables had a normal distribution (Supplementary file S2). Independent variables showing either positive or negative correlation of r ≥ 0.3 with the dependent variables were included in multiple linear regression analysis. For baseline trunk flexion strength, flexion co-activation index (CI%) (r = − 0.61 *p* = 0.002) was added to the model. For baseline extension strength, LES EMG amplitude (r = 0.60 *p* = 0.002) and disability (r = − 0.37 *p* = 0.054) were added to the model. For the follow-up measures of flexion strength, pain in the past week (r = − 0.41 *p* = 0.035), flexion CI% (r = − 0.51 *p* = 0.011), baseline trunk flexion strength (r = 0.83 *p* = 0.000), and average physical health (r = 0.43 *p* = 0.029) were added to the regression model. For follow-up extension strength data, LES EMG amplitude (r = 0.53 *p* = 0.008), and baseline trunk extension strength (r = 0.51 *p* = 0.010) were added to the regression model.

**Table 2:** Results of the univariate regression analyses between dependent variables and independent variables.

Covariates	Baseline		Follow up	
	Trunk flexion	Trunk extension	Trunk flexion	Trunk extension
Dependent variables				
Age	0.00 (0.499)	− 0.17 (0.235)	0.20 (0.199)	0.19 (0.203)
Sex	0.62 (0.002) **	0.65 (0.001) ***	0.75 (0.000) ***	0.65 (0.001) ***
Weight (kg)	0.48 (0.014) **	0.46 (0.021) *	0.51 (0.010) **	0.25 (0.136)
Independent variables (Baseline)				
Flex contraction (NPRS)	0.08 (0.369)	–	0.00 (0.490)	–
Ext contraction (NPRS)	–	0.10 (0.329)	–	− 0.12 (0.293)
Past week (NPRS)	− 0.34 (0.070)	− 0.35 (0.064)	− 0.41 (0.035) *	− 0.34 (0.066)
Pain 24h (NPRS)	− 0.14 (0.270)	− 0.26 (0.125)	− 0.18 (0.223)	− 0.17 (0.226)
RA EMG amplitude (RMS)	0.54 (0.006) **	–	0.41 (0.033) *	–
LES EMG amplitude (RMS)	–	0.60 (0.002) **	–	0.53 (0.008) **
LES-RA co-activation (Flex CI %)	− 0.61 (0.002) **	–	− 0.51 (0.011) **	–
RA-LES co-activation (Ext CI %)	–	− 0.33 (0.077)	–	− 0.13 (0.284)
Trunk flexion strength (N/m)	–	–	0.83 (0.000) ***	–
Trunk flexion change (N/m)	–	–	0.13 (0.287)	–
Trunk extension strength (N/m)	–	–	–	0.51 (0.010) **
Trunk extension change (N/m)	–	–	–	0.20 (0.188)
Endurance time (sec)	–	0.01 (0.478)	–	0.24 (0.146)
LES mean frequency (Hz)	–	− 0.07 (0.385)	–	− 0.06 (0.400)
Disability (ODI)	− 0.26 (0.134)	− 0.37 (0.054)	− 0.18 (0.220)	0.09 (0.345)
Kinesiophobia (TSK)	0.17 (0.228)	− 0.08 (0.369)	0.17 (0.234)	0.20 (0.191)
Average physical health (SF36)	0.19 (0.204)	0.32 (0.079)	0.43 (0.029) *	0.18 (0.213)
Limiting physical health (SF36)	− 0.04 (0.425)	0.20 (0.198)	− 0.14 (0.267)	− 0.17 (0.227)

Values are presented as correlation coefficient r (*p*-value), *P* = (*p ≤ 0.05, **(*p* > 0.001 to ≤ 0.01), ***p ≤ 0.001).

### Model results for independent outcomes

An overview of each final model is presented in Tables 3 and 4. Sex, age, and weight together explained 44% of the variance in maximum trunk flexion strength when measured at baseline. An additional 22% was explained by LES-RA co-activation, such that the final model significantly explained baseline flexion strength (F (4,15) = 7.53, P < 0.002, *R*^2^ = 0.66). For baseline extension strength, sex, age, weight, and LES EMG amplitude explained 65% of the variation. Regression of change and unstandardised regression coefficients (β) are presented in Table 3. Regression model performance outputs are presented in Supplementary file (S3).

**Table 3:** Final model to explain variation in baseline trunk flexion and extension strength.

	*R*^2^ change	β	*P*-value
Trunk flexion			
Covariates			
Age	.44	− .22	.82
Sex		19.35	.27
Weight		1.17	.11
Independent variables			
LES-RA co-activation	.22	− .13	.006
Model summary			
* R*^2^	.66		
F-change	F (4,15) = 7.53		
Significance of F	.002		
Trunk extension			
Covariates			
Age	.56	− 1.99	.233
Sex		27.68	.359
Weight		2.10	.072
Independent variables			
LES EMG amplitude	.09	3.38	.062
Model summary			
* R*^2^	.65		
F-change	F (4,15) = 7.152		
Significance of F	.002		

*R*^2^, coefficient of determination; B, unstandardized regression coefficients; LES, lumbar erector spinae; RA, rectus abdominus; B, unstandardized regression coefficients.

**Table 4:** Final model to explain variation in follow-up trunk flexion and extension strength.

	*R*^2^change	β	*P*-value
Trunk flexion			
Covariates			
Age	.58	1.36	.008
Sex		24.26	.008
Weight		− .15	.661
Independent variables			
LES-RA co-activation	.15	− .05	.440
Baseline flex strength	.10	.25	.054
SF36 physical function	.06	.54	.005
Past week pain (NPRS)	.04	− 4.03	.018
Model summary			
* R*^2^	.93		
F-change	F (7,12) = 23.68		
Significant of F	< .001		
Trunk extension			
Covariates			
Age	.52	2.65	.033
Sex		49.44	.030
Weight		− 1.07	.182
Independent variables			
LES EMG amplitude	.12	2.68	.038
Model summary			
* R*^2^	.64		
F-change	F (4,15) = 6.898		
Significant of F	.002		

*R*^2^, coefficient of determination; LES, lumbar erector spinae; RA, rectus abdominus; B, unstandardized regression coefficients; SF36, Short Form Health Survey; NPRS, numeric pain rating scale.

Covariates (sex, age, and weight) combined with LES-RA co-activation, baseline flexion strength, SF36 physical function subscale and the pain intensity over the past week explained 93% of the variation in the follow-up measure of trunk flexion strength (Table 4). Being an older male, with higher baseline trunk strength and higher physical function with less pain (over the past week) at baseline implied a greater increase in trunk flexion strength after the training intervention. In addition to sex, age, and weight, the LES EMG amplitude explained 12%, with the overall regression model predicting approximately 64% of the variance in trunk extension strength following the training intervention (*R*^2^ = 0.64, F (4,15) = 6.898, *p* = 0.002).

## Discussion

This study highlighted the importance of demographic characteristics for explaining variance in trunk isometric flexion and extension strength in participants with CLBP, both at baseline but also following 6 weeks of progressive resistance training. Additionally, the study showed that commonly evaluated PROMs (such as the level of disability, pain on exertion and kinesiophobia) did not explain the variance in baseline or post resistance training trunk strength.

The current study does not support reduced trunk strength being associated with kinesiophobia, measured using the TSK, in participants with CLBP^36^. The results of the present hierarchical regression analysis correspond well with the results from a previous study, which demonstrated a lack of association between kinesiophobiaand isometric trunk flexion and extension strength in individuals with CLBP^24^. However, the TSK is considered a generic measure of kinesiophobia^37^ and therefore, does not necessarily capture the fear associated with a specific task, such as the one tested herein. For this reason, task-specific psychological outcomes have been recommended to better predict the influence of psychological factors on physical performance in individuals with CLBP^23^. This is further supported by Matheve et al.^38^, who found that neither the TSK nor the total score of the photograph series of daily activities (PHODA-total) explained a significant portion of the variance in lumbar ROM during a lifting task. However, when the PHODA-lifting score, which depicts a person lifting a flowerpot with a bent back, was included in the model, it explained a significant 11% variance in lumbar ROM (*p* = 0.003). Another possible reason for these results could be that the testing positions and measured isometric efforts were not perceived as harmful and therefore, were not as fear-inducing compared to, for example, dynamic bending and lifting^39,40^.

In the present study, a weak correlation was observed between the average peak trunk flexion and extension torque and pain experienced during exertion (contraction pain intensity). This observation is supported by findings from previous research^41^, in which participants were asked to rate their anticipated pain prior to, and actual pain during, anisometric trunk extension task. The researchers concluded that the anticipation of pain, rather than the pain experienced during testing, was a significant predictor, explaining approximately 14–23% of the variation in trunk isometric strength among individuals with CLBP. In our sample, CLBP participants were classified as having only mild to moderate pain intensity (based on their NPRS score) which may not be enough to limit their ability to exert their maximum effort. Therefore, other pain-related psychological outcomes such as anticipation of pain, pain catastrophising and pain self-efficacy could have more of an influence on physical performance, including strength. These findings are supported by a recent systematic review and meta-analysis, in which, only one psychological factor, anticipated pain, had a moderate negative correlation with the maximal physical performance in individuals with CLBP^23^.

Our current study found a significant negative association between LES-RA co-activation and trunk strength, which influenced the variability of flexion strength both at baseline (*R*^2^ = 0.22) and after training (*R*^2^ = 0.15). However, the same was not true for extension strength in relation to the level of abdominal muscle co-activation. This finding is consistent with a previous investigation reporting higher thoracic and lumbar ES activation with more strenuous trunk flexion, while no difference in RA activity was observed during the trunk extension effort^42^. Similarly, biomechanical analysis reveals that co-contraction during isometric trunk flexion exertion was double the value of co-activation during extension exertion. Also, higher paraspinal muscle activity has been reported in healthy individuals during pushing tasks, possibly to maintain spinal stability^43^.

Differences in trunk muscle activity, including increased trunk flexors and extensors muscle co-activation, have been reported between individuals with CLBP and healthy individuals^44,45^. In the short term, increased trunk flexor and extensor muscle co-activation and paraspinal muscle activity, may enhance control of spinal motion (in this instance, limit spinal motion), as observed by Lee et al.^46^ and reduce associated symptom provocation. However, in the long term, that increased trunk muscle co-contraction could lead to increased spinal load and associated injury risk^47,48^. Increased antagonist muscle activity is supported by the “pain adaptation” model, which describes motor command adaptation in response to nociceptive input^49^. This leads to the facilitation of inhibitory pathways for agonist motor neurons and excitatory pathways for antagonist motor neurons^49^. This adaptive strategy aims to protect the spine from painful movements and maintain stability^50,51^. Antagonist co-activation served as a compensatory mechanism for muscle weakness as previously found in people recovered from LBP who were classified as weak compared to strong, showing higher EMG activation amplitude of trunk extensors with lower abdominal strength during controlled dynamic task^52^. A similar inverse correlation between antagonist activation and agonist isometric strength has been found in people with chronic neck pain^53^.

The relation between strength and absolute endurance, such as the number of repetitions performed at a given resistance, is well-established^54^. However, the current findings highlight the lack of correlation between the endurance time and isometric extension strength. The observed results are in agreement with previous studies showing a lack of correlation between extensor strength and endurance in individuals with CLBP^55^ and in asymptomatic individuals^56^. The lack of correlation observed in this study may be explained by the distinct physiological and biomechanical mechanisms underlying these two components of trunk muscle performance. Isometric strength reflects the maximum force-generating capacity of the muscle or group of muscles^57^, influenced by factors such as motor unit recruitment and neuromuscular activation, while endurance performance depends on the ability to sustain submaximal muscle activity or perform multiple repetitions^58^, influenced by metabolic efficiency and fatigue resistance. Additionally, the recruitment patterns during the Ito endurance test may further contribute to this lack of correlation as endurance time in the Ito test could be partially determined by the strength and endurance of the hip extensors rather than the lumbar extensors alone, due to the involvement of the hip extensors to maintain the required posture^59^. These findings suggest that strength and endurance capture different components of trunk muscle performance which might explain the lack of correlation in the current findings.

In this study, demographic variables, particularly age and sex, emerged as significant factors which could explain variation of trunk strength gain following resistance training in participants with CLBP, aligning with findings from a previous investigation measuring isometric^60^ and isokinetic trunk strength^61^. The magnitude of influence of sex (as indicated by the slope coefficient), reveals that males have follow-up flexion strength of 24.26 Nm and extension strength of 49.44 Nm, which is greater than females. This finding is consistent with earlier research indicating sex-based disparities in isometric and isokinetic strength abilities, with men exhibiting 1.5–2 times greater strength^62^. Such differences are attributed to the larger cross-sectional area and a higher distribution percentage of type II muscle fibres among men, which inherently possess a greater capacity for force and power generation^63^.

In the current study, pain experienced within the past week was one of the significant predictors (*p* = 0.018) of trunk flexion gain following 6 weeks of progressive resistance training. Previously, it has been found that improvement in pain intensity explained 20% improvement in trunk extension strength, as assessed using isokinetic dynamometer^28^. Apart from the pain experienced in the past week, none of the pain intensity measures displayed any correlation with trunk strength. Therefore, the evaluation of both sensory and cognitive dimensions of pain is important to gain a comprehensive understanding of its influence on physical functioning including strength.

The baseline trunk flexion strength explained 10% of the variability in follow-up trunk flexion strength. This finding is consistent with previous work, showing that a higher pre-operative strength level was associated with higher isometric flexion and extension strength 1 year after lumbar spine fusion^60^. In the present study, we found that LES EMG amplitude explained 9% and 12% of the variance in trunk extension strength at baseline and follow-up, respectively. This is not surprising as neuromuscular activation is one of the physiological determinants of muscle strength^64^.

### Clinical implications and future work

Trunk isometric strength is frequently used as an outcome measure in intervention studies. The findings of this study highlight the importance of individual factors, such as age, sex, baseline strength, and physical health, in explaining the variance in trunk strength changes. Specifically, older males with higher baseline strength and physical function, and lower pain levels, experienced greater gains in trunk flexion strength compared to their counterparts with lower baseline strength and physical function, and higher pain levels. These findings suggest that these factors my help clinicians better understand variations in outcomes following trunk-focused resistance training programmes. Moreover, the significant role of EMG parameters, including lumbar erector spinae (LES) amplitude and co-activation indices, in explaining baseline and follow-up strength outcomes suggests that neuromuscular function plays a critical role in trunk strength performance. This supports the integration of targeted neuromuscular training, such as exercises that enhance muscle activation and reduce antagonist co-activation, into rehabilitation programmes for CLBP.

The regression analysis in this study showed that neither the TSK nor ODI significantly explained variation in trunk isometric strength. Although it is commonly assumed that physical capacity might be influenced by kinesiophobia leading to disability^65^, as per the fear-avoidance model^66,67^, clinicians should be careful when drawing conclusions regarding patients’ physical capacity based solely on disability or pain-related fear scores. Instead of concentrating heavily on psychological barriers, programs could aim to improve physical capacity while fostering a sense of accomplishment and control over movement. This focus on enhancing self-efficacy and motivation is crucial, as it encourages patient adherence and long-term engagement with resistance training, thereby promoting sustained functional improvements. However, these assumptions need to be tested in further studies to explore the influence of other factors (e.g., pain perception, self-efficacy, pain tolerance and patient motivation) on trunk maximum isometric strength. Finally, while this study provides insights into factors explaining trunk strength improvement, it does not establish a direct link between strength gains and improvements in pain, health, or disability. Future research should explore these relationships to better understand the clinical relevance of trunk strength training in improving overall outcomes for individuals with CLBP.

### Methodological considerations

As the biopsychosocial nature of the CLBP condition is frequently emphasised^3^, one of the strengths of the current study is the inclusion of patient-reported and objective physical outcomes to explore their influence on trunk isometric muscle strength in participants with CLBP. Additionally, the influence of these outcomes on both baseline trunk muscle strength and changes in trunk muscle strength following an intervention was analysed.

However, our findings should be evaluated in light of the exploratory nature of our study, which has some limitations. Participants undergoing active LBP management were excluded to minimise the influence of ongoing treatments. However, some participants may have received prior LBP management, potentially influencing baseline trunk strength. For instance, recent strengthening exercises might have temporarily enhanced strength, whereas detraining or sedentary behaviour following pain management could have reduced strength. To minimise carryover effects, we implemented a 2-week washout period based on evidence that strength adaptations from training are generally preserved for less than 3 weeks before detraining effects occur^68,69^. While this approach aimed to reduce systematic bias, we acknowledge that prior pain management could still introduce random variability in baseline measures.

The sample size was calculated to ensure adequate power to evaluate the responsiveness of the hand-held dynamometer (HHD) in assessing trunk muscle strength in participants with CLBP. Given this focus, the sample may not have been sufficient to detect smaller, more nuanced effects of the other predictors included in our models. This presents a risk of both false positives and false negatives, particularly with the relatively high number of predictors in relation to the sample size. The limited sample size increases the chance that some relation between predictors and trunk muscle strength may have been identified as statistically significant purely by chance. This could lead to overestimating the influence of certain variables, particularly when there are multiple predictors competing for limited variance within a small sample (false positives). On the other hand, there may be meaningful associations between anthropometric or demographic variables and trunk muscle strength that were not detected (false negatives). These non-significant results may stem from the study being underpowered to detect smaller, yet clinically important, effects^70^.

Independent variables were first subjected to univariate analyses to identify their relationship with the response variable before being included in the multivariate model. This approach was complemented by checks for multicollinearity, including correlation analysis (cut-off < 0.7) and Variance Inflation Factor (VIF) assessments, both of which confirmed acceptable levels of multicollinearity among the selected variables. However, we acknowledge that this procedure may overlook interactions between variables and is less sophisticated than dimension reduction techniques, such as principal component analysis (PCA), particularly in studies with small sample sizes. Future research will consider adopting such techniques to further refine feature selection and model robustness. Additionally, the limited sample size poses challenges in terms of model performance. Given the number of predictors, there is a risk of overfitting, where the model captures idiosyncrasies of the sample rather than generalisable patterns. This overfitting can lead to inflated performance estimates within the study sample, but poorer performance when applied to new data^71^. For example, while demographic and anthropometric variables were included in the models based on theoretical relevance, they were not consistently significant across all models, possibly affecting the other factors’ weight in the models. The small sample size might undermine the robustness of the conclusion drawn and therefore results should be interpreted with caution.

To mitigate these issues, future research should aim to recruit larger sample sizes to reduce the risk of both false positives and negatives and to provide greater statistical power for detecting subtle but meaningful effects. This would also enhance the stability and generalisability of the models, ensuring more reliable conclusions regarding the factors influencing trunk muscle strength.

The participants were initially selected from the university population, demonstrating relatively good physical health, and experiencing mild to moderate levels of pain, disability, and kinesiophobia. Therefore, the findings of this study might not be broadly applicable to individuals more severely affected by CLBP.

## Conclusion

Our evaluation of factors which influence trunk muscle strength in participants with CLBP demonstrated that sex and age significantly influence the extent to which trunk muscle maximum isometric strength increases following resistance training but only trunk muscle EMG variables explained the variation in trunk muscle strength at baseline assessment. In this study, gains in flexion strength were explained by trunk muscle co-activation, past week pain (negatively correlated), baseline flexion strength, and baseline physical functioning (positively correlated). Only the LES EMG amplitude during maximum trunk extension explained the variance in trunk extension isometric strength at follow-up (positively correlated). Despite common assumptions, neither the disability level nor kinesiophobia significantly influenced trunk strength, indicating a necessity for further exploration into factors like self-efficacy, and patient motivation on maximum trunk strength.

## Materials and methods

### Study design and setting

This cross-sectional study is a secondary analysis of both baseline and follow-up data of participants included in a prospective cohort study evaluating the responsiveness of the HHD following 6 weeks of progressive resistance exercises in participants with CLBP. This study gained full ethical approval from the University of Birmingham ethics committee (ERN_22-0512), and all participants provide informed written consent before data collection. The study was conducted at laboratories within the CPR Spine, University of Birmingham, UK from September 2022 to July 2023 in accordance with the Declaration of Helsinki, and the current work is reported in accordance with the STrengthening the Reporting of OBservational studies in Epidemiology (STROBE) guidelines^72^ which is available in Supplementary file (S4).

### Participants

The study recruited participants with CLBP from the staff and student population of the University of Birmingham, United Kingdom via posters, emails, and word of mouth. Individuals aged 18–55 years, with a history of CLBP (i.e., pain lasting at least 3 months over the past 6 months^73^), pain intensity (i.e., ≥ 2 on a 0–10 numeric pain rating scale (NPRS)), and moderate disability (i.e., 20% on the Oswestry Disability Index (ODI)), were included. Due to due to the potential age-related changes of the musculoskeletal system with older age and the demands of repeated strength testing, we set the upper age limit to 55 years. This decision is supported by evidence of musculoskeletal decline with age documented in previous studies^74,75^. However, we acknowledge that other studies, such as one by Dallaway et al.^76^ tested maximal trunk strength in older adults above the age of 55, demonstrating that research in this area sometimes includes individuals beyond this age range.

Individuals with neurological and cardiovascular disorders were excluded. Additionally, those exhibiting signs of serious trauma, cancer, fractures, spinal stenosis, systemic or inflammatory conditions, or pregnancy were excluded. Participants were ineligible if they were undergoing active low back pain management at the time of data collection, participating in competitive athletics, or engaging in over 90 min of vigorous exercise daily. These criteria ensured a more homogenous sample and avoided the influence of treatment effects, enhanced athletic conditioning, or exercise-induced muscle adaptation from impacting the validity of the study findings.

### Protocol

Participants engaged in a six-week progressive resistance exercise programme, attending one session per week. Data were collected both at baseline and at a follow-up in the following sequence: (1) demographic information and PROMs, (2) maximum voluntary isometric contractions (MVICs) of trunk flexion and extension (direction randomized), (3) static endurance task (Ito test). (4) Simultaneous recordings of surface EMG from the rectus abdominis (RA) and lumbar erector spinae (LES) during the MVIC and the endurance task. Trunk strength was assessed using maximal isometric contractions due to their reliability in providing consistent and controlled measures of maximal strength, unaffected by factors such as speed or movement quality. While the progressive resistance training programme employed isotonic exercises to replicate functional, dynamic movements, studies have demonstrated that isotonic training contributes to overall muscular strength, including isometric strength, through neuromuscular adaptations^77–79^.

### Outcome measures

#### PROMs

Pain intensity was assessed using an NPRS^80^ to assess pain experienced during flexion and extension MVICs (contraction pain), average pain during the 24 h and average pain during the past week. LBP perceived disability was evaluated with the ODI with the total score expressed as a percentage^81^. General health assessed with the SF-36 (physical subscale only)^82^ and kinesiophobia evaluated with the 17-item TSK^83^. All of the aforementioned PROMs have established measurement properties, including reliability, validity, and responsiveness^82,84–86^. These properties support their use in this study to ensure accurate and meaningful assessment of participant outcomes.

#### Electromyography

High-density electromyography signals (HDEMG) were recorded from the LES bilaterally using 13 × 5 semi-disposable 2D electrode grids (OT Bioelettronica, Turin, Italy) of equally spaced electrodes (diameter: 3 mm with an interelectrode distance of 8 mm) which were placed 2 cm lateral to the L5 as described previously^87^. Before placement, the electrodes were prepared by attaching a double-side adhesive foam to the electrode surface (OT Bioelettronica, Italy), and to provide adequate electrode–skin contact, the electrode cavities were filled with highly conductive-adhesive paste (AC-CREAM, SPES Medica, Genoa, Italy).

Two surface bipolar EMG electrodes (WhiteSensor WS, Ambu A/S, Ballerup, Denmark; size: 36 × 40 mm, centre-to-centre distance approximately 3.6 cm) were placed over the RA bilaterally. Electrodes were positioned 3 cm laterally, halfway along the line between the xiphoid process and the umbilicus^88^, with reference electrodes placed at both anterior superior iliac spines, at C7 and wrist^89,90^. Before placing the electrodes, the participant’s skin was prepared by shaving if needed. Next, a gentle local abrasion was applied (Nuprep Skin Prep Gel; Weaver and Company, Aurora, USA) to lower skin impedance, followed by cleaning the area with water. Signals were amplified (Quattrocento-OT-Bioelettronica, Torino, Italy), and the collected torque signals from the isokinetic dynamometer (see details below) were synchronised with the EMG signals via the auxiliary input. All EMG signals were amplified by a factor of 150, sampled at 2048 Hz, automatically filtered with a second-order Butterworth band-pass filter (bandwidth: 10–350 Hz, first order, − 3 dB) and digitized with a 16-bit A/D converter. Signals recorded during the MVICs and during the endurance task were stored on a local computer using OT Biolab software (OT Bioelettronica, Turin, Italy) for further processing.

The recorded EMG signals were analysed offline, using custom scripts, (available in Supplementary file (S5). on MATLAB 2021a (The MathWorks Inc., USA). Fifty-nine differential EMG signals were obtained from the HDEMG electrode grids, formed by differentiating the 64 monopolar signals in the presumed direction of the muscle fibers^87^. Prior to signal analysis, a visual inspection was performed on all signals to eliminate channels containing noise or motion artefacts^91^. The rate of removal was less than 15%, ensuring that the EMG data could be accurately calculated and analysed^92^.

The EMG amplitude root mean square (RMS) in µV obtained during each MVICs were computed for every bipolar channel. For the LES, the average RMS across the 59 bipolar channels was then used. The RMS of LES and RA was used to calculate the co-activation index during the flexion and extension MVICs as follows:

$$coactivation\,index\,(CI\% ) = \frac{{EMG\,Antagonist}}{{EMG\,Agonist}} \times 100$$ ^93^

To assess myoelectric manifestations of muscle fatigue, the mean power spectral frequency (MNF) of the EMG signals were determined across the grid for the LES and the average determined during the endurance task^94^.

#### Maximum voluntary isometric contractions

**Fig. 1:**
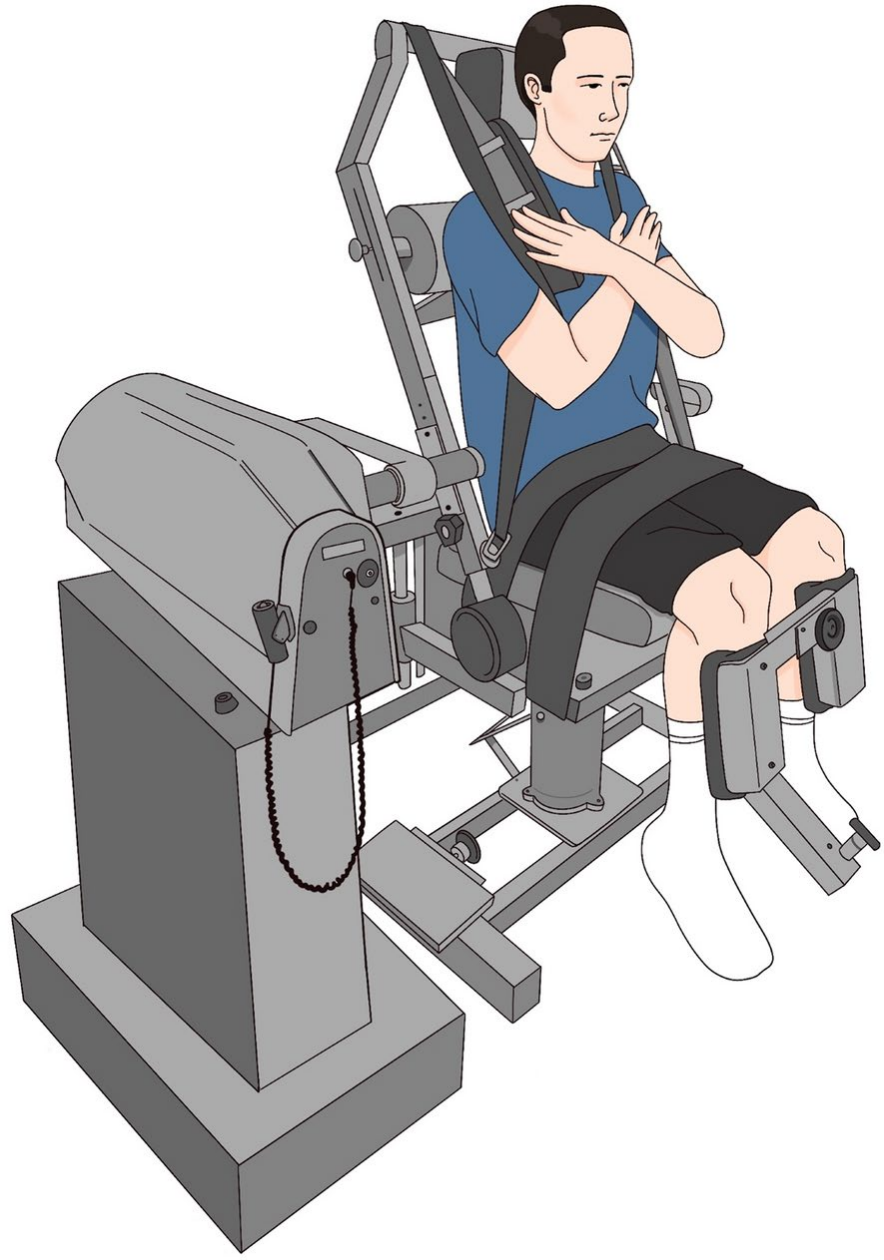
Trunk isometric strength (torque) measurement setup.

The MVICs were collected using the isokinetic dynamometer (System 3 Pro, Biodex Medical Systems, USA). Using the Biodex Dual Position back extension /flexion attachment, participants were seated on an adjustable seat in a compressed isolated lumbar position (approximately 15°) with both hips and knees in 90° flexion with feet parallel to the floor as recommended by the manufacturer^95^. This position was chosen to isolate of the lumbar area and allow for the proper HDEMG electrode placement^96^. For the measurement of trunk flexion and extension MVIC, the axis of dynamometer rotation was aligned with the participant’s anterior superior iliac spine, and the device was locked at 90° (50° according to the attachment’s goniometer). A scapular roll was positioned between the level of scapular spines and inferior angles. Participants were then secured in position using Velcro straps over the torso, hip, and thigh to isolate the trunk movement. The absolute peak torque in (Nm) of trunk flexion and extension was recorded during three MVICs, participants performed 1–2 submaximal familiarisation trials followed by three MVICs with 5-s hold and 2-min intervals in between trials and the mean of the three attempts was used for the analysis. The reliability of the MVIC measurements was assessed using the ICC to evaluate the consistency of performance across baseline and follow-up sessions, ensuring that the familiarisation process was sufficient. The mean value of the three MVIC attempts was used in the analysis as a measure of absolute torque, accounting for intra-trial variability and providing a stable, representative indicator of isometric trunk strength. Although the peak MVIC value is commonly used to reflect maximal capacity, averaging across trials mitigates the effects of natural variability in performance, such as fluctuations in effort, coordination, or transient fatigue, resulting in a more robust and reliable measure. Additionally, previous reports of average and peak MVCs indicate that average MVC has higher correlation with pain and disability in individuals with CLBP^97^. Pain intensity was verbally rated by the participants using (0–10) NPRS after each MVIC trial. The mean contraction pain was then taken for further analysis. Trunk isometric strength measurement setup is highlighted in (Fig. 1).

#### Endurance task

To complete the endurance task, an isometric back endurance test (Ito test) was conducted as originally described by Ito et al.^98^. The participants lie prone on the plinth without any fixation to minimize the activation of hip extensors^99^, and to minimise the lumbar lordosis, a semi-circular foam pad (18 cm diameter) was positioned across the anterior pelvis at the level of the ASIS. Participants were asked to lift the sternum off the plinth raising their upper body by ~ 15°. While maintaining this position for 300 s or to task failure (deviation in the trunk angle by more than 10° at any point). Prior to the task, the examiner gave standardised instructions and explained the accurate procedure to the participants, then participants were given a short familiarisation trial of 5 s to ensure they had the correct technique.

Throughout the task, the trunk orientation was monitored both visually and using the accelerometer of a smartphone (iPhone 11 Pro Max) which has been shown to provide valid and reliable kinematic results^100,101^. The smartphone was fixed using double-sided adhesive tape below the T4 level, and the trunk orientation data was collected using the MATLAB Mobile app (MathWorks, Natick, US) installed on the smartphone. The smartphone position was standardized (landscape orientation, screen facing up, the camera on the low right corner) so that changes in trunk acceleration and orientation would be displayed in real-time using a custom script on MATLAB 2019a (The MathWorks Inc., USA). The script allowed for setting a specific target trunk angle of 15°, with upper and lower thresholds of 10° (i.e., ± 10° from the target). This real-time feedback ensured participants maintained the desired trunk position throughout the endurance task.

**Fig. 2:**
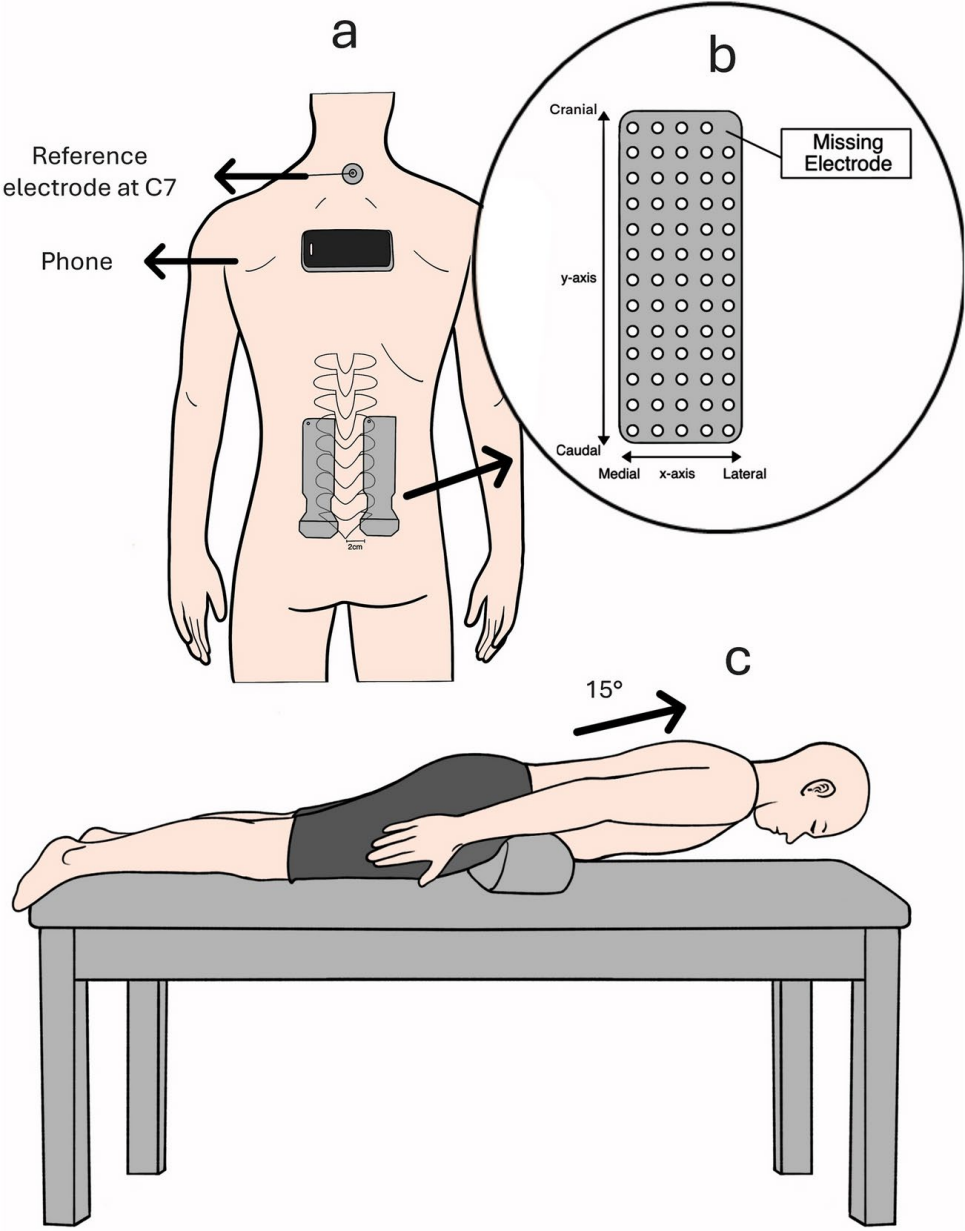
Illustration of the endurance task setup: (**a**) The approximate positioning of the HDEMG grids which was 2 cm lateral to L5 spinous process and the phone placement. (**b**) Layout of the electrode grid showing the x and y-axes. (**c**) The participant position for the Ito endurance test.

While performing the endurance task, participants were timed using a stopwatch. Verbal encouragement was consistently given to participants, and they were informed about the duration they had maintained the contraction. The endurance task setup is highlighted in (Fig. 2).

### Progressive resistance training

A functional dynamometer (Primus, BTE Technologies, USA) was used to perform progressive isotonic resistance exercises targeting the trunk muscles. These exercises were performed while seated on the Primus chair, with straps secured around the thighs and pelvis to minimise their involvement^102,103^. Exercises included trunk flexion, extension, and right and left rotation. The inclusion of rotational trunk movements in the resistance training programme aimed to promote the comprehensive development of core muscles critical for functional trunk stability and daily activities by challenging the core muscles in multiple planes and ranges of movement, as supported by^104^. Before commencing the training program, participants underwent three MVIC trials for each movement in the mid-position. This was done to determine the appropriate training load.

Over 6 weeks, participants performed supervised progressive resistance exercises performed at the laboratory once per week^105,106^. The same assessor trained all participants during these training sessions, which lasted 1 h each. The sessions began with a 10-min general warm-up, followed by tailored warm-up exercises for the four specific exercises. These warm-up sets consisted of one set of 10 repetitions at 50% of the training load^107^, followed by two sets of resistance exercises with one-minute rest intervals^108^. The resistance load was adjusted at 50–80% of the individual maximum effort (i.e., MVIC), with (8–15 reps/set) as recommended for individuals with CLBP^106^. The load increased by 5% in each session, taking into account the participant’s ability to safely and effectively perform the exercises. Isotonic (concentric/eccentric) mode was used for training, with participants taking two seconds to perform concentric movements, holding for approximately one second, and then four seconds for eccentric movements^78^. The average baseline and follow-up training load progression can be found in Supplementary Material (S6). Each session concluded with a 10-min cool-down, including static stretching exercises for the back and abdominal muscles. They were not encouraged to perform any other exercises for their trunk besides those performed at the weekly training sessions.

### Statistical analysis

Statistical analysis was performed using SPSS Statistics version 28 (IBM, USA). A hierarchical multiple regression analyses was conducted to evaluate the factors that explain variation of baseline trunk muscle strength and variation of the absolute difference in trunk flexion and extension MVICs following the training programme.

Measures of pain intensity, disability, kinesiophobia, and SF-36 (specifically, the physical functioning subscale) were evaluated as independent variables. Additionally, PerFOMs such as RA EMG amplitude (µV) during MVIC, LES EMG amplitude (µV) during MVIC, total endurance time (sec), antagonist-agonist co-activation during MVICs, and the average MNF (Hz) recorded from the LES during the endurance task were taken into consideration. Baseline flexion and extension strength levels were incorporated as independent variables to investigate the strength changes after the intervention.

Independent variables were entered into the hierarchical multiple regression model which was conducted in two stages. Firstly, each independent variable was entered into a univariate regression analysis. In the second stage, only variables showing either positive or negative correlation of ≥ 0.3 with a *p*-value of < 0.05 were included to evaluate the relation between the dependent and independent variables. A hierarchical regression model was then applied and only variables increasing the overall model *R*^2^ were included. To ensure the validity of the regression model, several assumptions were rigorously tested: (1) Linearity was assessed by examining scatterplots of each predictor against the dependent variable. (2) Univariate and multivariate outliers were identified using boxplots, while multivariate outliers were assessed through checking the Cook’s distance to detect any data points significantly deviating from the multivariate distribution. (3) Normality of residuals was evaluated by inspecting histogram and the P–P plot. (4) Multicollinearity of predictors was examined through the correlation matrix to ensure no excessively high correlations between independent variables, correlation of > 0.7 either positive or negative^109^. Additionally, VIF values were calculated, with a threshold of VIF < 10 used to identify and avoid multicollinearity issues. Homoscedasticity was checked by plotting residuals against predicted values to confirm that the variance of residuals remained constant across all levels of the independent variables (approximately constantly spread). These steps ensured that the assumptions of the regression model were met, providing confidence in the validity of the results^110,111^.

Data are reported as mean (standard deviation) for continuous data, the coefficient of determination (*R*^2^) and regression constant. Considering the potential influence of age, sex and weight on physical performance^112^, these variables were treated as covariates. Consequently, irrespective of their statistical significance, they were consistently retained in the model. All PROMs and PerFOMs were included as independent variables for each task (flexion and extension) for both baseline and follow-up trunk strength measurements. Variables including contraction pain, mean EMG amplitude and co-activation index were considered as independent variables for the corresponding dependent variable e.g. flexion contraction pain, RA EMG amplitude, and LES-RA co-activation (CI%) were included for both baseline and follow-up analysis of flexion strength data. The average MNF (Hz) of the EMG signal recorded from LES during the endurance task was included as a predictive variable for the trunk extension strength analysis.

## Supplementary Information

Supplementary Information.

## Data Availability

The datasets generated and/or analysed during the current study are available upon reasonable request. Requests for access to the data should be addressed to corresponding author. Access to the data will be provided in accordance with applicable data protection and privacy regulations.
