# Supplementary material for: Explaining trunk strength variation and improvement following resistance training in people with chronic low back pain: clinical and performance-based outcomes analysis
Source: Sci Rep. 2025 Mar 13;15:8657. doi: 10.1038/s41598-025-93280-2 (PMC11906778; doi:10.1038/s41598-025-93280-2)

Supplementary material 1:

Reliability

Intraclass Correlation Coefficient (ICC) Results for M VIC Reliability Across Sessions

Case Processing Summary

		N	%
Cases	Valid	20	100.0
	Excluded[a]	0	.0
	Total	20	100.0

a. Listwise deletion based on all variables in the procedure.

Reliability Statistics

Cronbach's Alpha	Cronbach's Alpha Based on Standardized Items	N of Items
.650	.678	2

Item Statistics

	Mean	Std. Deviation	N
Baseline_Extension	99.5580	59.05322	20
FollowUp_Extension	108.8459	41.12482	20

Inter-Item Correlation Matrix

	Baseline_Extension	FollowUp_Extension
Baseline_Extension	1.000	.513
FollowUp_Extension	.513	1.000

Summary Item Statistics

	Mean	Minimum	Maximum	Range	Maximum / Minimum	Variance	N of Items
Item Means	104.202	99.558	108.846	9.288	1.093	43.132	2

Scale Statistics

Mean	Variance	Std. Deviation	N of Items
208.4039	7669.340	87.57477	2

Intraclass Correlation Coefficient

	Intraclass Correlation[b]	95% Confidence Interval		F Test with True Value 0		
		Lower Bound	Upper Bound	Value	df1	df2
Single Measures	.481[a]	.061	.756	2.853	19	19
Average Measures	.650[c]	.115	.861	2.853	19	19

Intraclass Correlation Coefficient

	F Test with ..
	Sig
Single Measures	.014
Average Measures	.014

Two-way mixed effects model where people effects are random and measures effects are fixed.

a. The estimator is the same, whether the interaction effect is present or not.

b. Type C intraclass correlation coefficients using a consistency definition. The between-measure variance is excluded from the denominator variance.

c. This estimate is computed assuming the interaction effect is absent, because it is not estimable otherwise.

Reliability

Scale: ALL VARIABLES

Case Processing Summary

		N	%
Cases	Valid	20	100.0
	Excluded[a]	0	.0
	Total	20	100.0

a. Listwise deletion based on all variables in the procedure.

Reliability Statistics

Cronbach's Alpha	Cronbach's Alpha Based on Standardized Items	N of Items
.903	.909	2

Item Statistics

	Mean	Std. Deviation	N
Baseline_Flexion	84.7328	39.40843	20
FollowUp_Flexion	99.9244	33.53077	20

Inter-Item Correlation Matrix

	Baseline_Flexion	FollowUp_Flexion
Baseline_Flexion	1.000	.834
FollowUp_Flexion	.834	1.000

Summary Item Statistics

	Mean	Minimum	Maximum	Range	Maximum / Minimum	Variance	N of Items
Item Means	92.329	84.733	99.924	15.192	1.179	115.392	2

Scale Statistics

Mean	Variance	Std. Deviation	N of Items
184.6572	4880.569	69.86107	2

Intraclass Correlation Coefficient

	Intraclass Correlation[b]	95% Confidence Interval		F Test with True Value 0		
		Lower Bound	Upper Bound	Value	df1	df2
Single Measures	.823[a]	.606	.926	10.294	19	19
Average Measures	.903[c]	.755	.962	10.294	19	19

Intraclass Correlation Coefficient

	F Test with ..
	Sig
Single Measures	<.001
Average Measures	<.001

Two-way mixed effects model where people effects are random and measures effects are fixed.

a. The estimator is the same, whether the interaction effect is present or not.

b. Type C intraclass correlation coefficients using a consistency definition. The between-measure variance is excluded from the denominator variance.

c. This estimate is computed assuming the interaction effect is absent, because it is not estimable otherwise.

Supplementary material 2:

Dependent variables normality test

Case Processing Summary

	Cases					
	Valid		Missing		Total	
	N	Percent	N	Percent	N	Percent
Baseline_Flexion	20	100.0%	0	0.0%	20	100.0%
Baseline_Extension	20	100.0%	0	0.0%	20	100.0%
FollowUp_Flexion	20	100.0%	0	0.0%	20	100.0%
FollowUp_Extension	20	100.0%	0	0.0%	20	100.0%

Descriptives

			Statistic	Std. Error
Baseline_Flexion	Mean		84.7328	8.81199
	95% Confidence Interval for Mean	Lower Bound	66.2891	
		Upper Bound	103.1765	
	5% Trimmed Mean		84.4522	
	Median		77.4561	
	Variance		1553.024	
	Std. Deviation		39.40843	
	Minimum		22.28	
	Maximum		152.24	
	Range		129.96	
	Interquartile Range		45.08	
	Skewness		.471	.512
	Kurtosis		-.551	.992
Baseline_Extension	Mean		99.5580	13.20470
	95% Confidence Interval for Mean	Lower Bound	71.9203	
		Upper Bound	127.1958	
	5% Trimmed Mean		96.4632	
	Median		89.1886	
	Variance		3487.283	
	Std. Deviation		59.05322	
	Minimum		8.55	
	Maximum		246.27	
	Range		237.72	
	Interquartile Range		90.14	
	Skewness		.785	.512
	Kurtosis		.447	.992
FollowUp_Flexion	Mean		99.9244	7.49771
	95% Confidence Interval for Mean	Lower Bound	84.2315	
		Upper Bound	115.6173	
	5% Trimmed Mean		100.0183	

Descriptives

			Statistic	Std. Error
	Median		92.6754	
	Variance		1124.312	
	Std. Deviation		33.53077	
	Minimum		45.00	
	Maximum		153.16	
	Range		108.16	
	Interquartile Range		55.76	
	Skewness		.249	.512
	Kurtosis		-1.046	.992
FollowUp_Extension	Mean		108.8459	9.19579
	95% Confidence Interval for Mean	Lower Bound	89.5989	
		Upper Bound	128.0929	
	5% Trimmed Mean		107.4829	
	Median		107.4123	
	Variance		1691.251	
	Std. Deviation		41.12482	
	Minimum		41.26	
	Maximum		200.96	
	Range		159.70	
	Interquartile Range		57.04	
	Skewness		.470	.512
	Kurtosis		-.149	.992

Tests of Normality

	Kolmogorov-Smirnov[a]			Shapiro-Wilk		
	Statistic	df	Sig.	Statistic	df	Sig.
Baseline_Flexion	.125	20	.200*	.929	20	.150
Baseline_Extension	.125	20	.200*	.953	20	.414
FollowUp_Flexion	.129	20	.200*	.946	20	.305
FollowUp_Extension	.119	20	.200*	.970	20	.763

*. This is a lower bound of the true significance.

a. Lilliefors Significance Correction

Baseline_Flexion

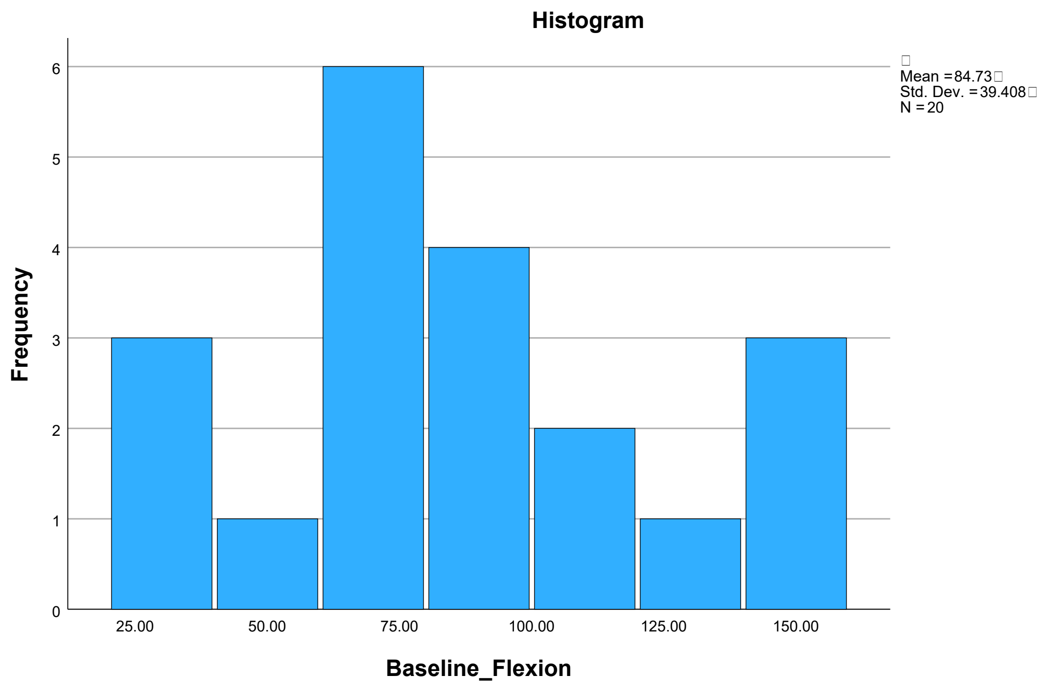
Histogram
Mean =84.73
Std. Dev. =39.408
N =20
Frequency
0
1
2
3
4
5
6
25.00
50.00
75.00
100.00
125.00
150.00
Baseline_Flexion

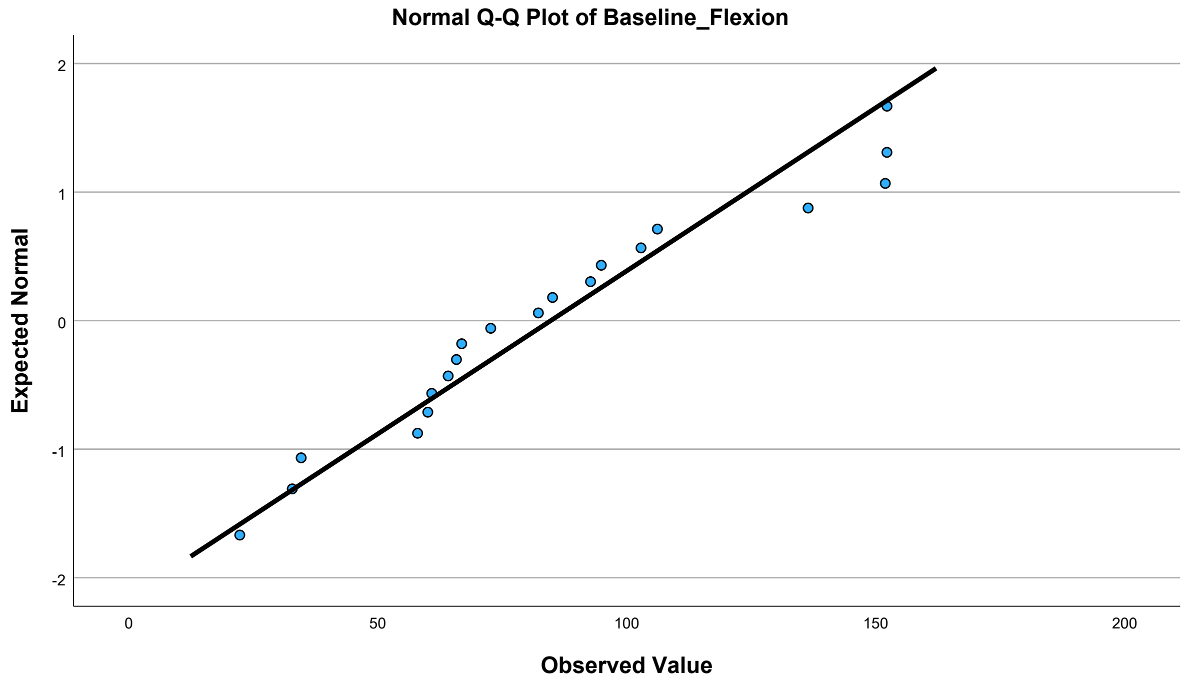
Normal Q-Q Plot of Baseline_Flexion
Expected Normal
2
1
0
-1
-2
0
50
100
150
200
Observed Value

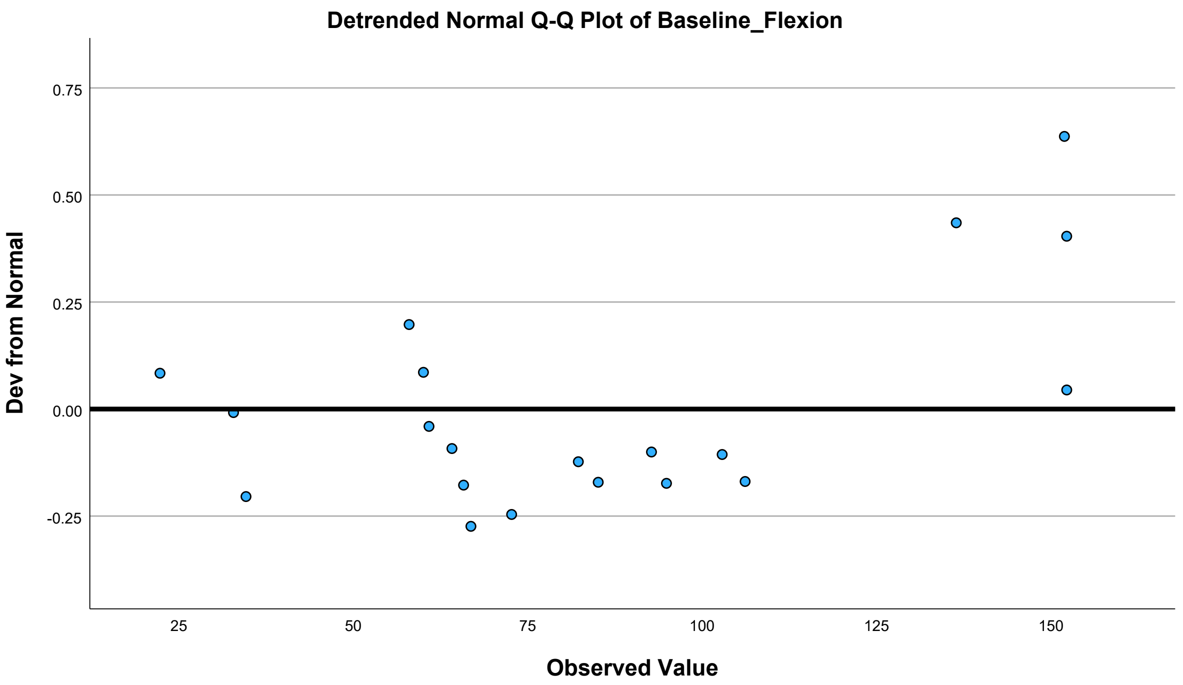

Baseline_Extension

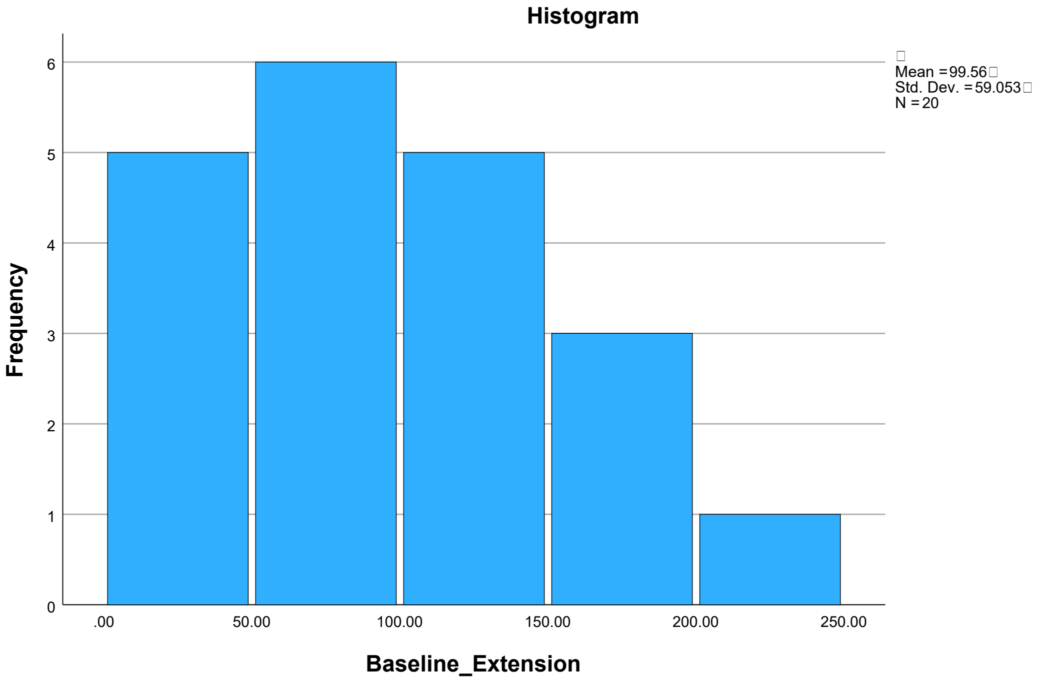

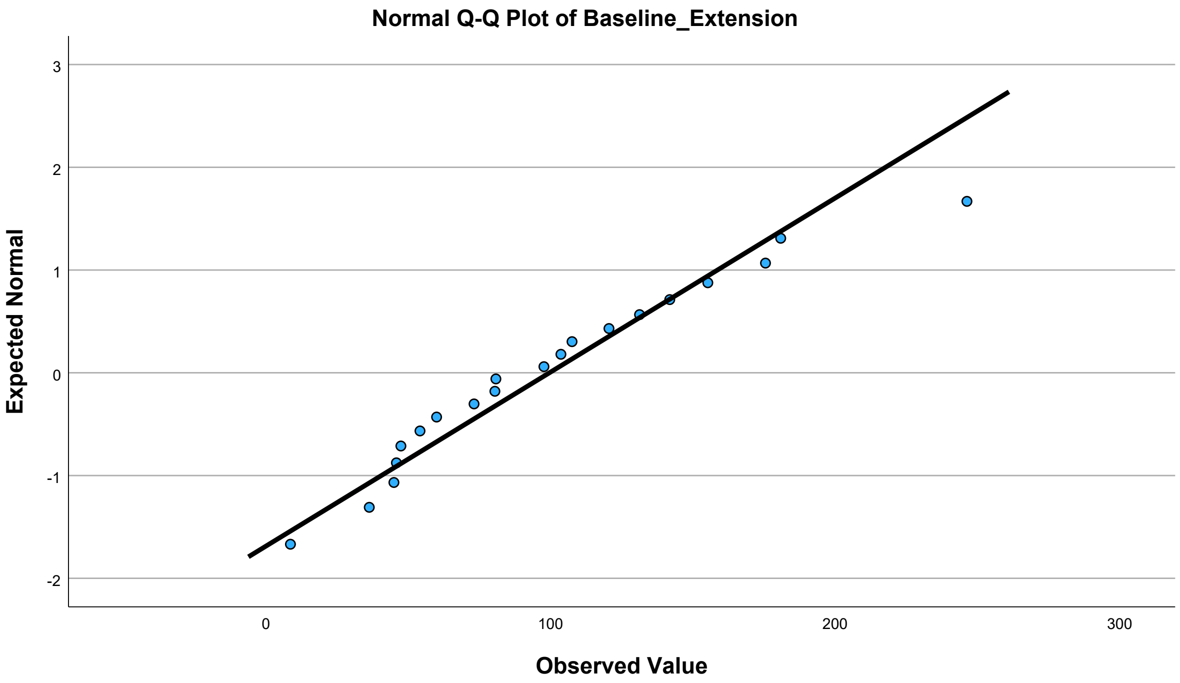

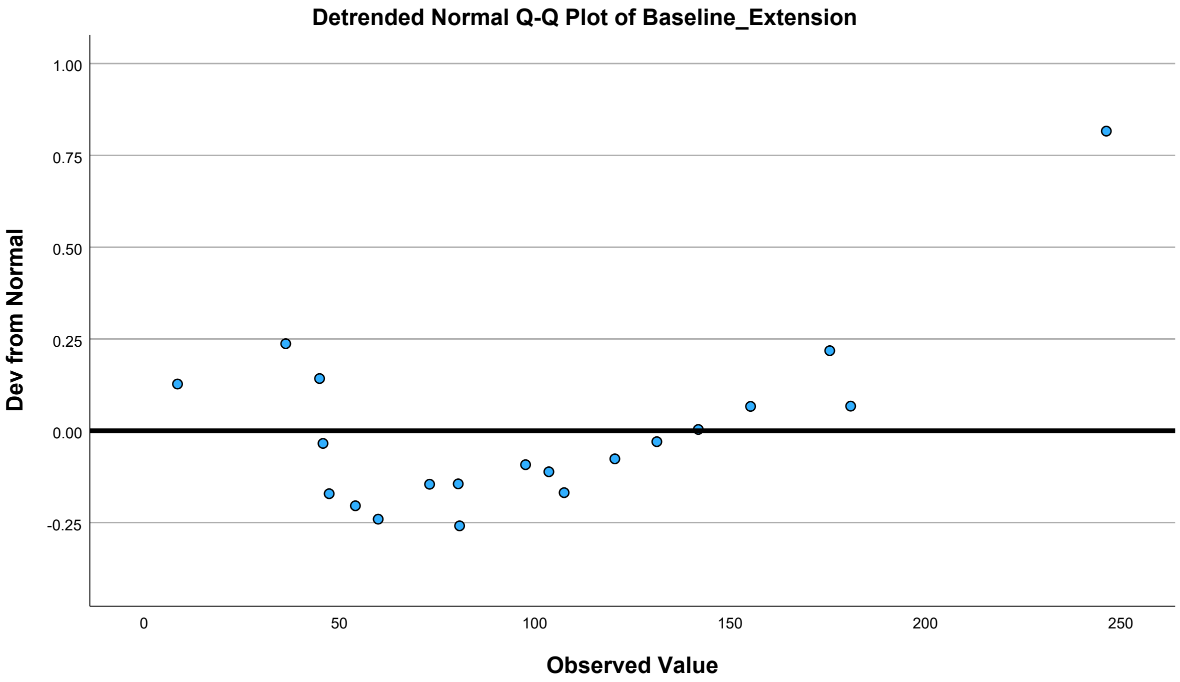

FollowUp_Flexion

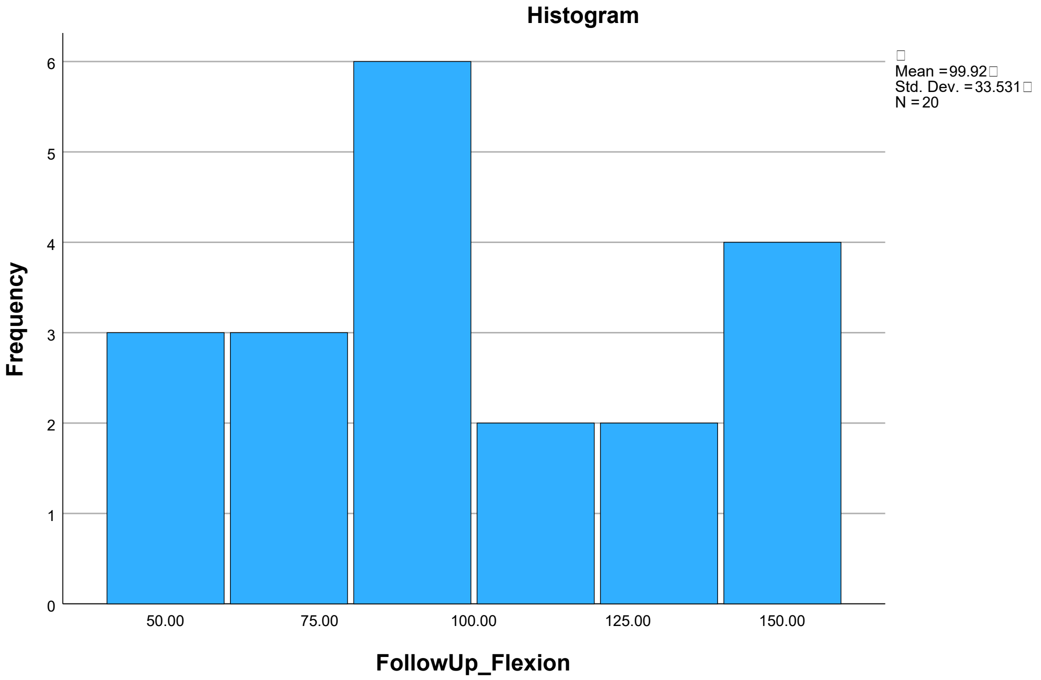
Histogram
Mean =99.92
Std. Dev. =33.531
N =20
Frequency
0
1
2
3
4
5
6
50.00
75.00
100.00
125.00
150.00
FollowUp_Flexion

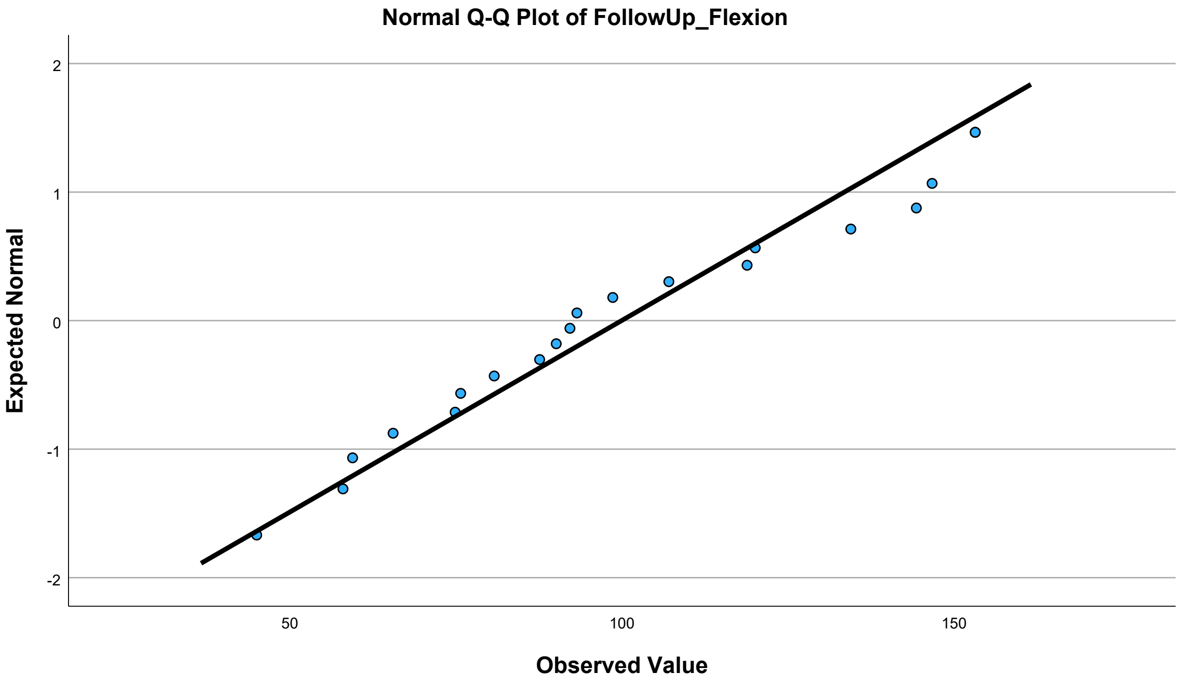
Normal Q-Q Plot of FollowUp_Flexion
Expected Normal
2
1
0
-1
-2
50
100
150
Observed Value

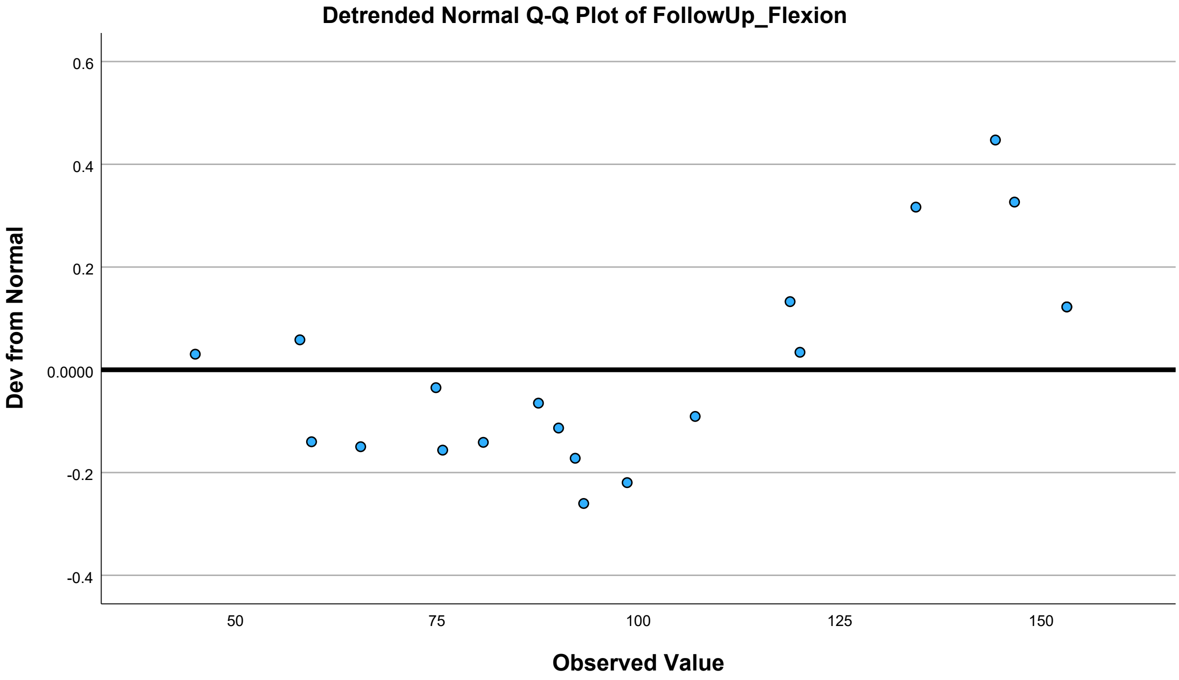

FollowUp_Extension

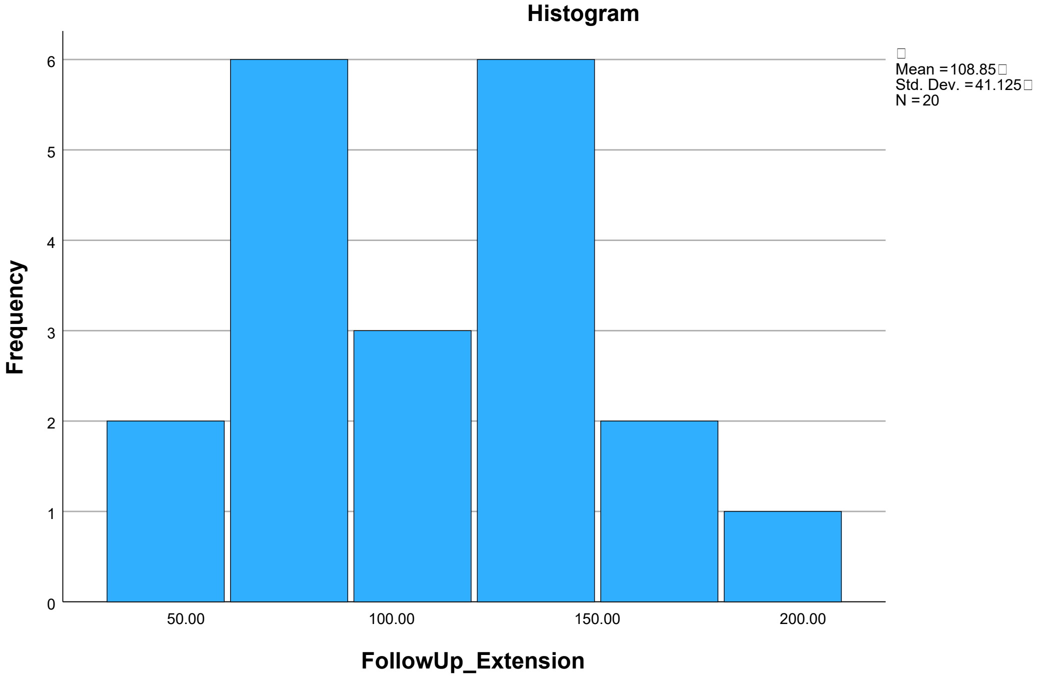

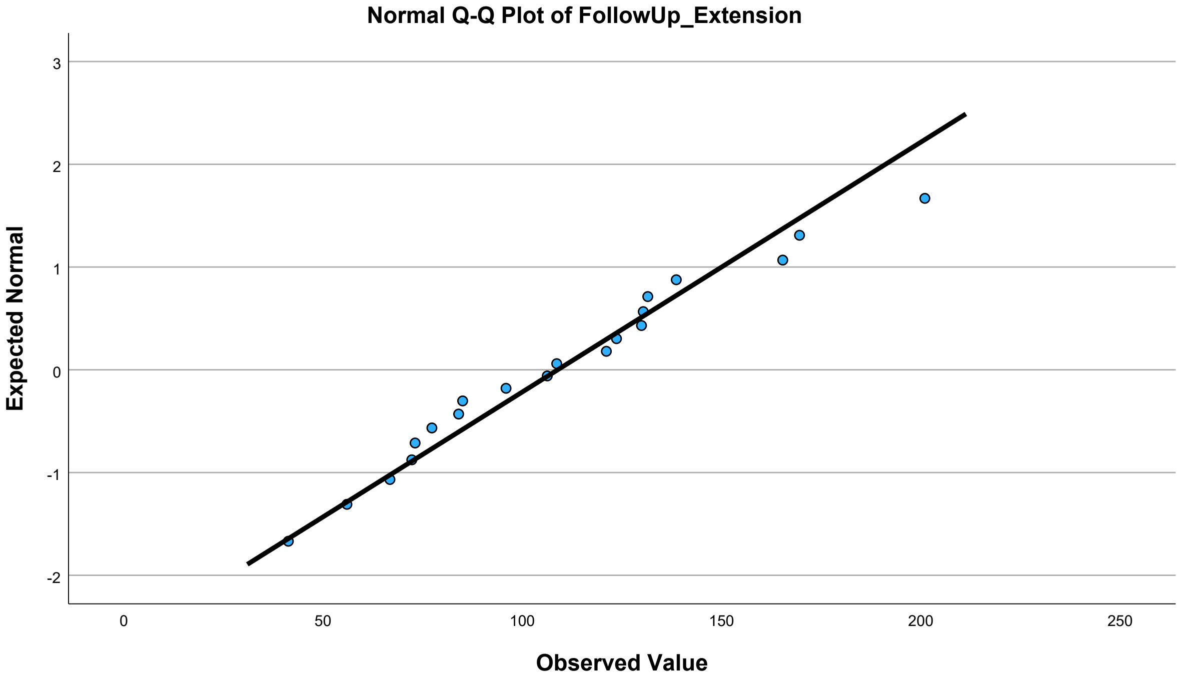
Normal Q-Q Plot of FollowUp_Extension
Expected Normal
Observed Value
3
2
1
0
-1
-2
0
50
100
150
200
250

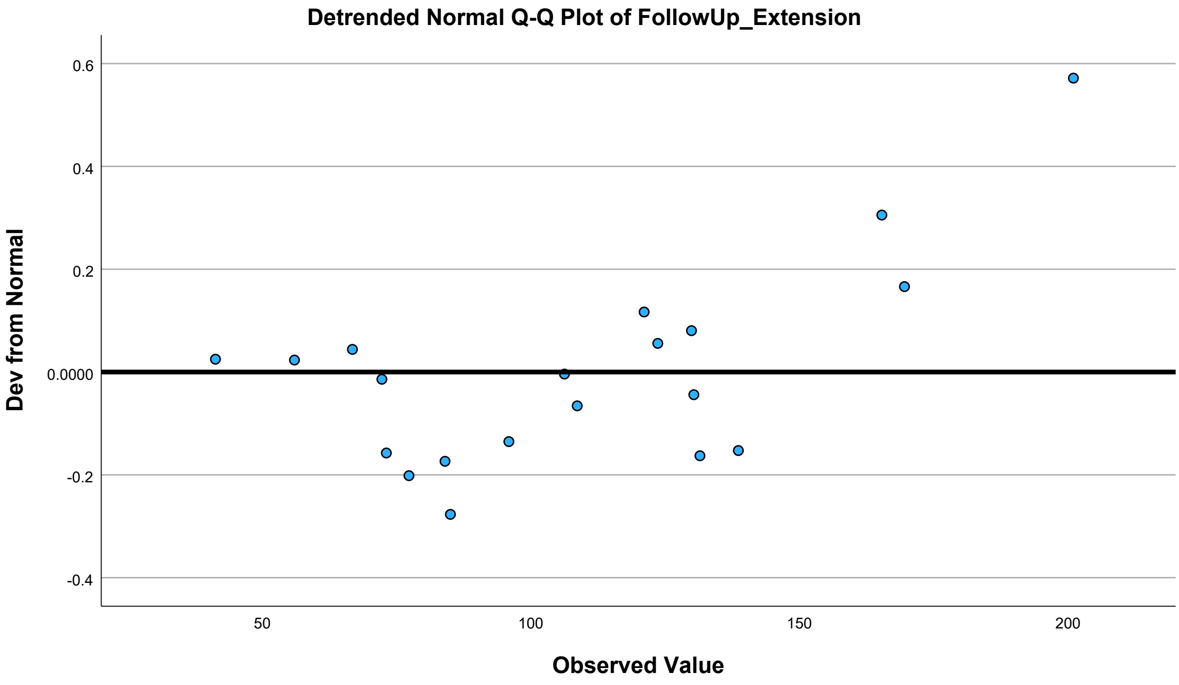
Detrended Normal Q-Q Plot of FollowUp_Extension
Dev from Normal
Observed Value
0.6
0.4
0.2
0.0000
-0.2
-0.4
50
100
150
200

Supplementary material 3:

Regression

Descriptive Statistics

	Mean	Std. Deviation	N
Baseline_Extension_Torque	99.5581	59.05347	20
Gender	.3500	.48936	20
Age_years	33.2500	7.35473	20
Weight_kg	70.8000	12.72213	20
Ext_MeanEMG_Amplitude	18.4968	7.30763	20

Correlations

		Baseline_Extension_Torque	Gender	Age_years	Weight_kg
Pearson Correlation	Baseline_Extension_Torque	1.000	.655	-.171	.461
	Gender	.655	1.000	.165	.637
	Age_years	-.171	.165	1.000	.493
	Weight_kg	.461	.637	.493	1.000
	Ext_MeanEMG_Amplitude	.607	.422	-.440	-.040
Sig. (1-tailed)	Baseline_Extension_Torque	.	<.001	.235	.021
	Gender	.001	.	.244	.001
	Age_years	.235	.244	.	.014
	Weight_kg	.021	.001	.014	.
	Ext_MeanEMG_Amplitude	.002	.032	.026	.433
N	Baseline_Extension_Torque	20	20	20	20
	Gender	20	20	20	20
	Age_years	20	20	20	20
	Weight_kg	20	20	20	20
	Ext_MeanEMG_Amplitude	20	20	20	20

Correlations

		Ext_MeanEMG_Amplitude
Pearson Correlation	Baseline_Extension_Torque	.607
	Gender	.422
	Age_years	-.440
	Weight_kg	-.040
	Ext_MeanEMG_Amplitude	1.000
Sig. (1-tailed)	Baseline_Extension_Torque	.002
	Gender	.032
	Age_years	.026
	Weight_kg	.433
	Ext_MeanEMG_Amplitude	.
N	Baseline_Extension_Torque	20
	Gender	20
	Age_years	20
	Weight_kg	20
	Ext_MeanEMG_Amplitude	20

Variables Entered/Removed[a]

Model	Variables Entered	Variables Removed	Method
1	Weight_kg, Age_years, Gender[b]	.	Enter
2	Ext_MeanEMG_Amplitude[b]	.	Enter

a. Dependent Variable: Baseline_Extension_Torque

b. All requested variables entered.

Model Summary[c]

Model	R	R Square	Adjusted R Square	Std. Error of the Estimate	Change Statistics R Square Change	F Change
1	.750[a]	.563	.481	42.54320	.563	6.870
2	.810[b]	.656	.564	38.98047	.093	4.058

Model Summary[c]

Model	Change Statistics df1	df2	Sig. F Change	Durbin-Watson
1	3	16	.003	
2	1	15	.062	2.657

a. Predictors: (Constant), Weight_kg, Age_years, Gender

b. Predictors: (Constant), Weight_kg, Age_years, Gender, Ext_MeanEMG_Amplitude

c. Dependent Variable: Baseline_Extension_Torque

ANOVA[a]

Model		Sum of Squares	df	Mean Square	F	Sig.
1	Regression	37300.153	3	12433.384	6.870	.003[b]
	Residual	28958.785	16	1809.924		
	Total	66258.938	19			
2	Regression	43466.780	4	10866.695	7.152	.002[c]
	Residual	22792.157	15	1519.477		
	Total	66258.938	19			

a. Dependent Variable: Baseline_Extension_Torque

b. Predictors: (Constant), Weight_kg, Age_years, Gender

c. Predictors: (Constant), Weight_kg, Age_years, Gender, Ext_MeanEMG_Amplitude

Coefficients[a]

Model		Unstandardized Coefficients B	Std. Error	Standardized Coefficients Beta	t
1	(Constant)	76.783	66.917		1.147
	Gender	60.394	26.554	.500	2.274
	Age_years	-3.430	1.564	-.427	-2.193
	Weight_kg	1.634	1.158	.352	1.411
2	(Constant)	-55.636	89.889		-.619
	Gender	27.687	29.250	.229	.947
	Age_years	-1.990	1.602	-.248	-1.242
	Weight_kg	2.104	1.086	.453	1.937
	Ext_MeanEMG_Amplitude	3.389	1.682	.419	2.015

Coefficients[a]

Model		Sig.	95.0% Confidence Interval for B Lower Bound	95.0% Confidence Interval for B Upper Bound	Correlations Zero-order	Correlations Partial
1	(Constant)	.268	-65.075	218.641		
	Gender	.037	4.102	116.686	.655	.494
	Age_years	.043	-6.747	-.114	-.171	-.481
	Weight_kg	.177	-.820	4.088	.461	.333
2	(Constant)	.545	-247.230	135.957		
	Gender	.359	-34.657	90.031	.655	.237
	Age_years	.233	-5.404	1.425	-.171	-.305
	Weight_kg	.072	-.211	4.419	.461	.447
	Ext_MeanEMG_Amplitude	.062	-.197	6.975	.607	.461

Coefficients[a]

Model		Correlations Part	Collinearity Statistics Tolerance	Collinearity Statistics VIF
1	(Constant)			
	Gender	.376	.564	1.773
	Age_years	-.362	.720	1.390
	Weight_kg	.233	.439	2.278
2	(Constant)			
	Gender	.143	.390	2.562
	Age_years	-.188	.576	1.736
	Weight_kg	.293	.419	2.388
	Ext_MeanEMG_Amplitude	.305	.529	1.890

a. Dependent Variable: Baseline_Extension_Torque

Excluded Variables[a]

Model		Beta In	t	Sig.	Partial Correlation	Collinearity . Tolerance
1	Ext_MeanEMG_Amplitude	.419[b]	2.015	.062	.461	.529

Excluded Variables[a]

Model		Collinearity Statistics VIF	Collinearity Statistics Minimum Tolerance
1	Ext_MeanEMG_Amplitude	1.890	.390

a. Dependent Variable: Baseline_Extension_Torque

b. Predictors in the Model: (Constant), Weight_kg, Age_years, Gender

Collinearity Diagnostics[a]

Model	Dimension	Eigenvalue	Condition Index	Variance Proportions			
				(Constant)	Gender	Age_years	Weight_kg
1	1	3.440	1.000	.00	.02	.00	.00
	2	.528	2.552	.00	.57	.00	.00
	3	.023	12.187	.30	.00	.85	.02
	4	.009	19.861	.70	.41	.14	.97
2	1	4.323	1.000	.00	.01	.00	.00
	2	.529	2.858	.00	.40	.00	.00
	3	.127	5.832	.00	.03	.04	.01
	4	.014	17.529	.06	.01	.91	.33
	5	.006	26.561	.94	.55	.05	.67

Collinearity Diagnostics[a]

Model	Dimension	Variance ...
		Ext_MeanEMG_ Amplitude
1	1	
	2	
	3	
	4	
2	1	.00
	2	.00
	3	.38
	4	.21
	5	.41

a. Dependent Variable: Baseline_Extension_Torque

Residuals Statistics[a]

	Minimum	Maximum	Mean	Std. Deviation	N
Predicted Value	14.6454	185.9646	99.5581	47.83017	20
Std. Predicted Value	-1.775	1.807	.000	1.000	20
Standard Error of Predicted Value	11.756	27.167	19.038	4.282	20
Adjusted Predicted Value	-5.7381	182.0771	97.7626	50.54152	20
Residual	-60.33901	60.30735	.00000	34.63506	20
Std. Residual	-1.548	1.547	.000	.889	20
Stud. Residual	-1.744	1.861	.020	1.028	20
Deleted Residual	-76.58437	88.08118	1.79552	46.56515	20
Stud. Deleted Residual	-1.887	2.051	.026	1.082	20
Mahal. Distance	.778	8.278	3.800	2.073	20
Cook's Distance	.000	.328	.071	.090	20
Centered Leverage Value	.041	.436	.200	.109	20

a. Dependent Variable: Baseline_Extension_Torque

Charts

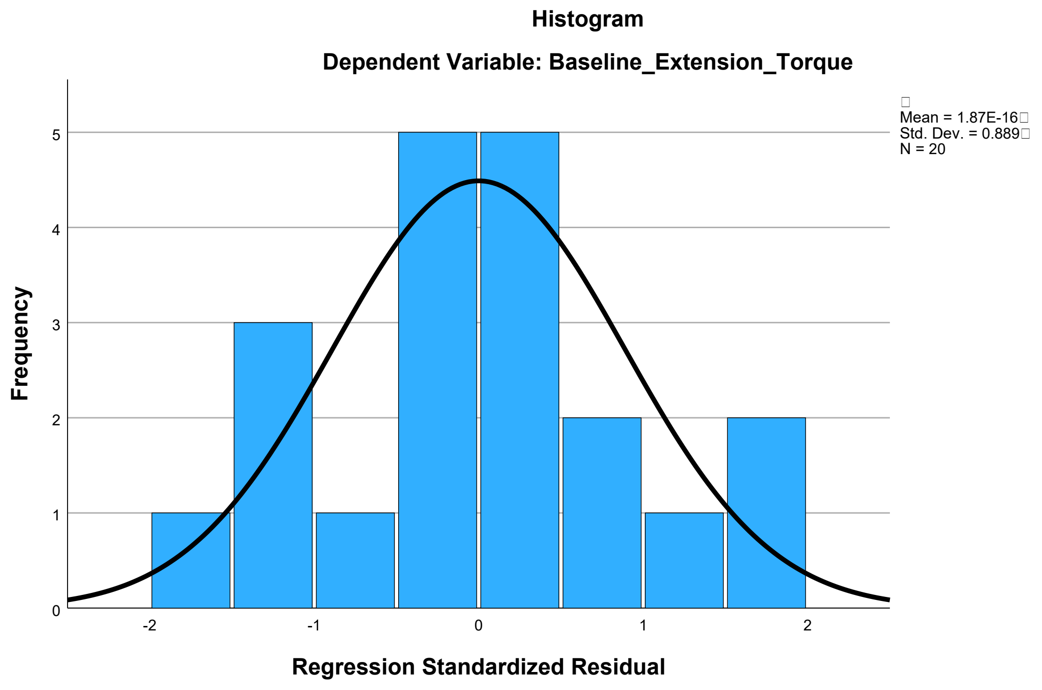

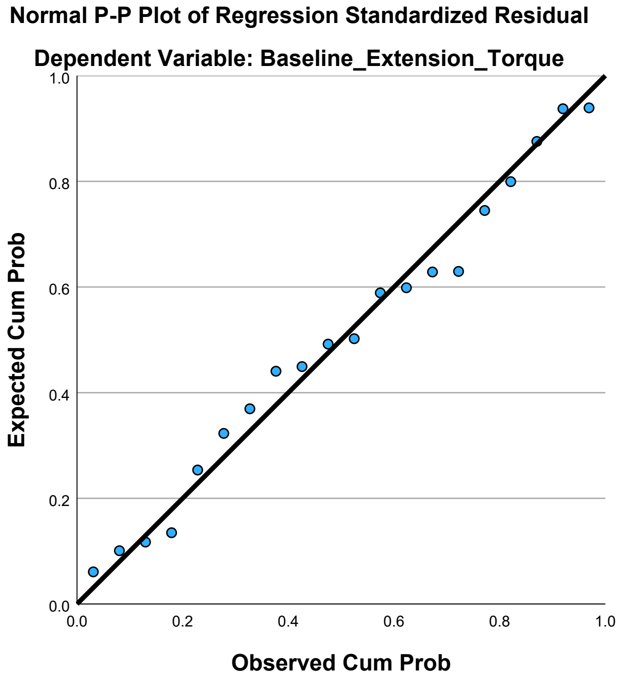

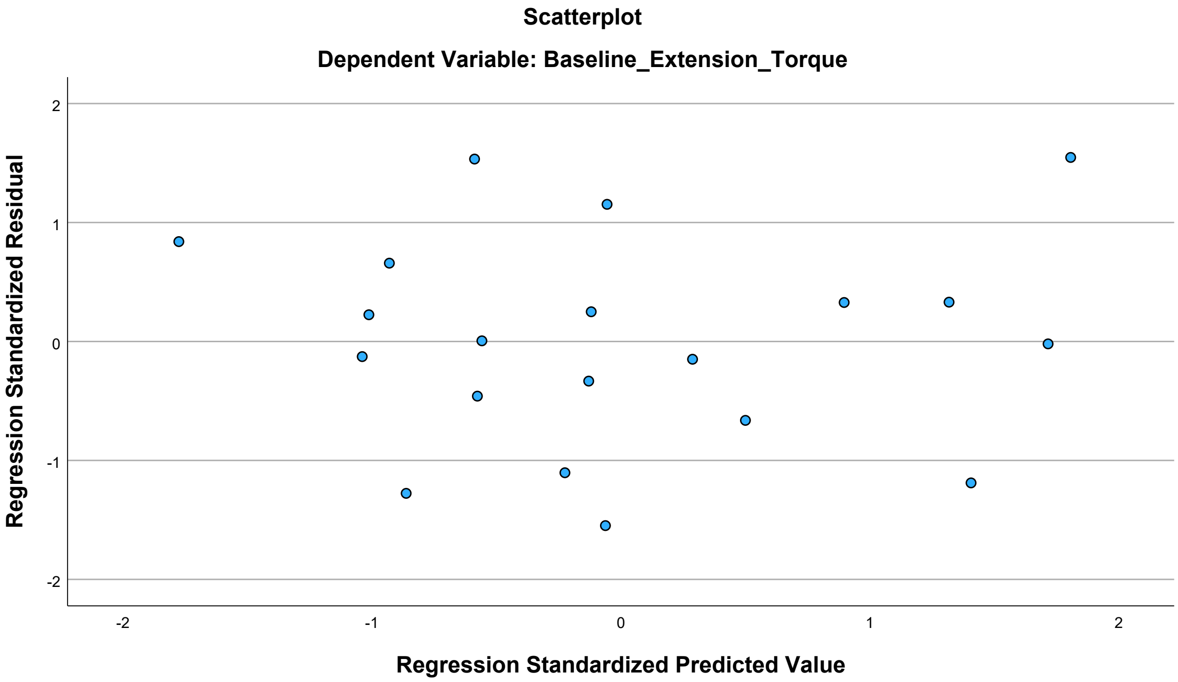

Your license will expire in 11 days.

Regression

Descriptive Statistics

	Mean	Std. Deviation	N
Baseline_Flexion_Nm	84.7328	39.40843	20
Age_years	33.2500	7.35473	20
Gender	1.3500	.48936	20
Weight_kg	70.8000	12.72213	20
SMEAN(Coactivation_Flex)	180.5984	158.72698	20

Correlations

		Baseline_Flexion_Nm	Age_years	Gender	Weight_kg
Pearson Correlation	Baseline_Flexion_Nm	1.000	.000	.623	.489
	Age_years	.000	1.000	.165	.493
	Gender	.623	.165	1.000	.637
	Weight_kg	.489	.493	.637	1.000
	SMEAN(Coactivation_Flex)	-.610	.337	-.271	.040
Sig. (1-tailed)	Baseline_Flexion_Nm	.	.499	.002	.014
	Age_years	.499	.	.244	.014
	Gender	.002	.244	.	.001
	Weight_kg	.014	.014	.001	.
	SMEAN(Coactivation_Flex)	.002	.073	.123	.433
N	Baseline_Flexion_Nm	20	20	20	20
	Age_years	20	20	20	20
	Gender	20	20	20	20
	Weight_kg	20	20	20	20
	SMEAN(Coactivation_Flex)	20	20	20	20

Correlations

		SMEAN (Coactivation_Flex)
Pearson Correlation	Baseline_Flexion_Nm	-.610
	Age_years	.337
	Gender	-.271
	Weight_kg	.040
	SMEAN(Coactivation_Flex)	1.000
Sig. (1-tailed)	Baseline_Flexion_Nm	.002
	Age_years	.073
	Gender	.123
	Weight_kg	.433
	SMEAN(Coactivation_Flex)	.
N	Baseline_Flexion_Nm	20
	Age_years	20
	Gender	20
	Weight_kg	20
	SMEAN(Coactivation_Flex)	20

Variables Entered/Removed[a]

Model	Variables Entered	Variables Removed	Method
1	Weight_kg, Age_years, Gender[b]	.	Enter
2	SMEAN (Coactivation_Flex)[b]	.	Enter

a. Dependent Variable: Baseline_Flexion_Nm

b. All requested variables entered.

Model Summary[c]

Model	R	R Square	Adjusted R Square	Std. Error of the Estimate	Change Statistics	
					R Square Change	F Change
1	.663[a]	.439	.334	32.16271	.439	4.175
2	.817[b]	.668	.579	25.56595	.229	10.322

Model Summary[c]

Model	Change Statistics			Durbin-Watson
	df1	df2	Sig. F Change	
1	3	16	.023	
2	1	15	.006	2.561

a. Predictors: (Constant), Weight_kg, Age_years, Gender

b. Predictors: (Constant), Weight_kg, Age_years, Gender, SMEAN(Coactivation_Flex)

c. Dependent Variable: Baseline_Flexion_Nm

ANOVA[a]

Model		Sum of Squares	df	Mean Square	F	Sig.
1	Regression	12956.428	3	4318.809	4.175	.023[b]
	Residual	16551.036	16	1034.440		
	Total	29507.464	19			
2	Regression	19703.196	4	4925.799	7.536	.002[c]
	Residual	9804.269	15	653.618		
	Total	29507.464	19			

a. Dependent Variable: Baseline_Flexion_Nm

b. Predictors: (Constant), Weight_kg, Age_years, Gender

c. Predictors: (Constant), Weight_kg, Age_years, Gender, SMEAN(Coactivation_Flex)

Coefficients[a]

Model		Unstandardized Coefficients		Standardized Coefficients	
		B	Std. Error	Beta	t
1	(Constant)	7.753	44.540		.174
	Age_years	-1.209	1.183	-.226	-1.022
	Gender	37.622	20.075	.467	1.874
	Weight_kg	.938	.875	.303	1.071
2	(Constant)	7.462	35.405		.211
	Age_years	-.225	.989	-.042	-.227
	Gender	19.353	16.940	.240	1.142
	Weight_kg	1.173	.700	.379	1.677
	SMEAN(Coactivation_Flex)	-.135	.042	-.546	-3.213

Coefficients[a]

Model		Sig.	95.0% Confidence Interval for B		Correlations
			Lower Bound	Upper Bound	Zero-order
1	(Constant)	.864	-86.667	102.174	
	Age_years	.322	-3.716	1.298	.000
	Gender	.079	-4.935	80.179	.623
	Weight_kg	.300	-.918	2.793	.489
2	(Constant)	.836	-68.002	82.925	
	Age_years	.823	-2.332	1.883	.000
	Gender	.271	-16.755	55.460	.623
	Weight_kg	.114	-.318	2.665	.489
	SMEAN(Coactivation_Flex)	.006	-.225	-.046	-.610

Coefficients[a]

Model		Correlations: Partial	Correlations: Part	Collinearity Statistics: Tolerance	Collinearity Statistics: VIF
1	(Constant)				
	Age_years	-.248	-.191	.720	1.390
	Gender	.424	.351	.564	1.773
	Weight_kg	.259	.201	.439	2.278
2	(Constant)				
	Age_years	-.059	-.034	.650	1.537
	Gender	.283	.170	.501	1.998
	Weight_kg	.397	.250	.434	2.303
	SMEAN(Coactivation_Flex)	-.638	-.478	.768	1.302

a. Dependent Variable: Baseline_Flexion_Nm

Excluded Variables[a]

Model		Beta In	t	Sig.	Partial Correlation	Collinearity . Tolerance
1	SMEAN(Coactivation_Flex)	-.546[b]	-3.213	.006	-.638	.768

Excluded Variables[a]

Model		Collinearity Statistics: VIF	Collinearity Statistics: Minimum Tolerance
1	SMEAN(Coactivation_Flex)	1.302	.434

a. Dependent Variable: Baseline_Flexion_Nm

b. Predictors in the Model: (Constant), Weight_kg, Age_years, Gender

Collinearity Diagnostics[a]

Model	Dimension	Eigenvalue	Condition Index	Variance Proportions: (Constant)	Age_years	Gender	Weight_kg
1	1	3.893	1.000	.00	.00	.00	.00
	2	.076	7.173	.03	.09	.57	.00
	3	.022	13.323	.62	.68	.03	.00
	4	.010	20.031	.35	.23	.40	1.00
2	1	4.516	1.000	.00	.00	.00	.00
	2	.398	3.367	.00	.00	.02	.00
	3	.055	9.053	.08	.10	.58	.00
	4	.021	14.562	.56	.72	.01	.00
	5	.010	21.627	.35	.18	.39	.99

Collinearity Diagnostics[a]

Model	Dimension	Variance ... SMEAN (Coactivation_Flex)
1	1	
	2	
	3	
	4	
2	1	.01
	2	.65
	3	.30
	4	.04
	5	.01

a. Dependent Variable: Baseline_Flexion_Nm

Residuals Statistics[a]

	Minimum	Maximum	Mean	Std. Deviation	N
Predicted Value	30.6206	140.9510	84.7328	32.20264	20
Std. Predicted Value	-1.680	1.746	.000	1.000	20
Standard Error of Predicted Value	7.348	19.062	12.402	3.180	20
Adjusted Predicted Value	33.5936	145.2537	85.1905	33.69909	20
Residual	-36.36940	45.44076	.00000	22.71594	20
Std. Residual	-1.423	1.777	.000	.889	20
Stud. Residual	-1.665	1.957	-.009	1.040	20
Deleted Residual	-50.38531	61.94611	-.45764	31.71393	20
Stud. Deleted Residual	-1.781	2.191	-.002	1.098	20
Mahal. Distance	.620	9.613	3.800	2.475	20
Cook's Distance	.000	.581	.087	.147	20
Centered Leverage Value	.033	.506	.200	.130	20

a. Dependent Variable: Baseline_Flexion_Nm

Charts

Histogram

Dependent Variable: Baseline_Flexion_Nm

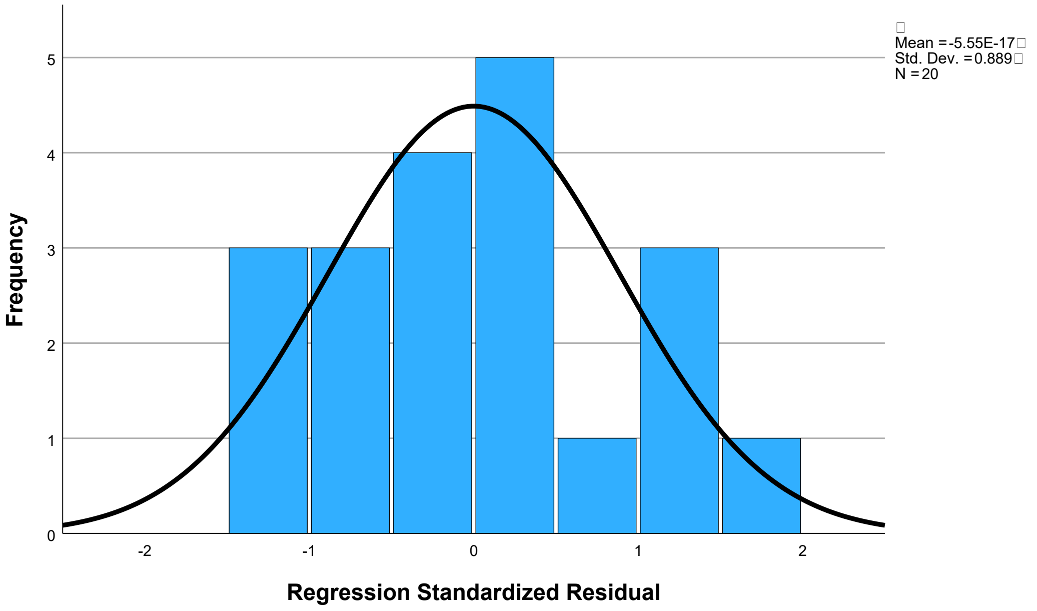

Normal P-P Plot of Regression Standardized Residual

Dependent Variable: Baseline_Flexion_Nm

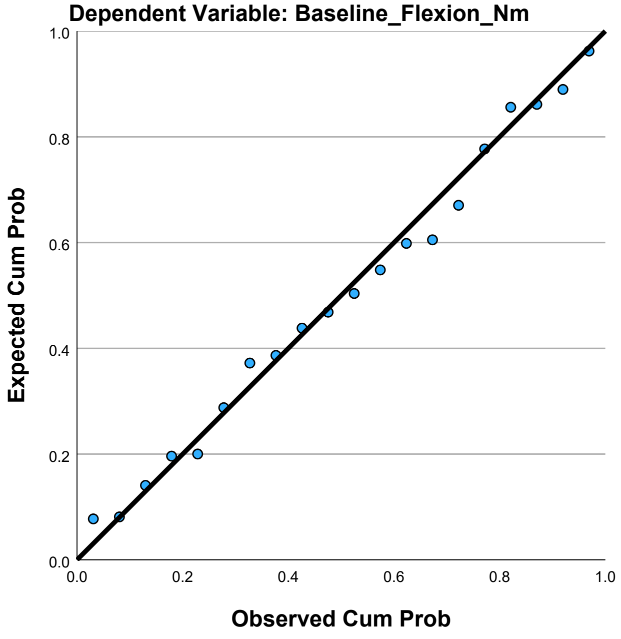

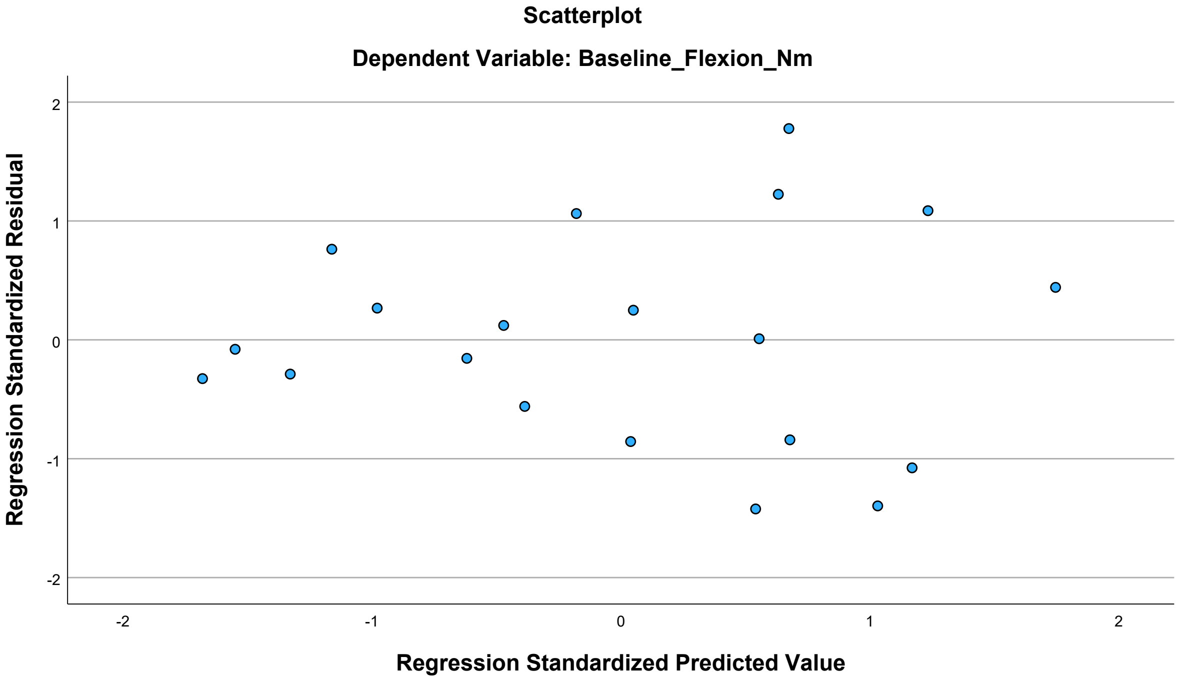
Scatterplot
Dependent Variable: Baseline_Flexion_Nm
Regression Standardized Residual
Regression Standardized Predicted Value
2
1
0
-1
-2
-2
-1
0
1
2

Regression

Descriptive Statistics

	Mean	Std. Deviation	N
FollowUp_Extension_Torque	108.8459	41.12482	20
Gender	.3500	.48936	20
Age_years	33.2500	7.35473	20
Weight_kg	70.8000	12.72213	20
SMEAN (Ext_MeanEMG_Amplitude)	18.4968	7.30763	20

Correlations

		FollowUp_Extension_Torque	Gender	Age_years	Weight_kg
Pearson Correlation	FollowUp_Extension_Torque	1.000	.657	.197	.258
	Gender	.657	1.000	.165	.637
	Age_years	.197	.165	1.000	.493
	Weight_kg	.258	.637	.493	1.000
	SMEAN (Ext_MeanEMG_Amplitude)	.531	.422	-.440	-.040
Sig. (1-tailed)	FollowUp_Extension_Torque	.	<.001	.203	.136
	Gender	.001	.	.244	.001
	Age_years	.203	.244	.	.014
	Weight_kg	.136	.001	.014	.
	SMEAN (Ext_MeanEMG_Amplitude)	.008	.032	.026	.433
N	FollowUp_Extension_Torque	20	20	20	20
	Gender	20	20	20	20
	Age_years	20	20	20	20
	Weight_kg	20	20	20	20
	SMEAN (Ext_MeanEMG_Amplitude)	20	20	20	20

Correlations

		SMEAN (Ext_MeanEMG_Amplitude)
Pearson Correlation	FollowUp_Extension_Torque	.531
	Gender	.422
	Age_years	-.440
	Weight_kg	-.040
	SMEAN (Ext_MeanEMG_Amplitude)	1.000
Sig. (1-tailed)	FollowUp_Extension_Torque	.008
	Gender	.032
	Age_years	.026
	Weight_kg	.433
	SMEAN (Ext_MeanEMG_Amplitude)	.
N	FollowUp_Extension_Torque	20
	Gender	20
	Age_years	20
	Weight_kg	20
	SMEAN (Ext_MeanEMG_Amplitude)	20

Variables Entered/Removed[a]

Model	Variables Entered	Variables Removed	Method
1	Weight_kg, Age_years, Gender[b]	.	Enter
2	SMEAN (Ext_MeanEMG_Amplitude)[b]	.	Enter

a. Dependent Variable: FollowUp_Extension_Torque

b. All requested variables entered.

Model Summary[c]

Model	R	R Square	Adjusted R Square	Std. Error of the Estimate	Change Statistics	
					R Square Change	F Change
1	.726[a]	.527	.438	30.82276	.527	5.941
2	.805[b]	.648	.554	27.46782	.121	5.147

Model Summary[c]

Model	Change Statistics			Durbin-Watson
	df1	df2	Sig. F Change	
1	3	16	.006	
2	1	15	.038	2.037

a. Predictors: (Constant), Weight_kg, Age_years, Gender

b. Predictors: (Constant), Weight_kg, Age_years, Gender, SMEAN(Ext_MeanEMG_Amplitude)

c. Dependent Variable: FollowUp_Extension_Torque

ANOVA[a]

Model		Sum of Squares	df	Mean Square	F	Sig.
1	Regression	16933.083	3	5644.361	5.941	.006[b]
	Residual	15200.680	16	950.042		
	Total	32133.763	19			
2	Regression	20816.542	4	5204.136	6.898	.002[c]
	Residual	11317.221	15	754.481		
	Total	32133.763	19			

a. Dependent Variable: FollowUp_Extension_Torque

b. Predictors: (Constant), Weight_kg, Age_years, Gender

c. Predictors: (Constant), Weight_kg, Age_years, Gender, SMEAN (Ext_MeanEMG_Amplitude)

Coefficients[a]

Model		Unstandardized Coefficients B	Unstandardized Coefficients Std. Error	Standardized Coefficients Beta	t
1	(Constant)	134.644	48.482		2.777
	Gender	75.397	19.238	.897	3.919
	Age_years	1.507	1.133	.270	1.330
	Weight_kg	-1.445	.839	-.447	-1.723
2	(Constant)	29.560	63.341		.467
	Gender	49.442	20.611	.588	2.399
	Age_years	2.650	1.129	.474	2.348
	Weight_kg	-1.072	.765	-.332	-1.400
	SMEAN (Ext_MeanEMG_Amplitude)	2.689	1.185	.478	2.269

Coefficients[a]

Model		Sig.	95.0% Confidence Interval for B Lower Bound	95.0% Confidence Interval for B Upper Bound	Correlations Zero-order
1	(Constant)	.013	31.867	237.421	
	Gender	.001	34.613	116.180	.657
	Age_years	.202	-.896	3.910	.197
	Weight_kg	.104	-3.223	.333	.258
2	(Constant)	.647	-105.448	164.567	
	Gender	.030	5.511	93.373	.657
	Age_years	.033	.244	5.056	.197
	Weight_kg	.182	-2.703	.560	.258
	SMEAN (Ext_MeanEMG_Amplitude)	.038	.163	5.216	.531

Coefficients[a]

Model		Correlations Partial	Correlations Part	Collinearity Statistics Tolerance	Collinearity Statistics VIF
1	(Constant)				
	Gender	.700	.674	.564	1.773
	Age_years	.315	.229	.720	1.390
	Weight_kg	-.396	-.296	.439	2.278
2	(Constant)				
	Gender	.527	.368	.390	2.562
	Age_years	.518	.360	.576	1.736
	Weight_kg	-.340	-.215	.419	2.388
	SMEAN (Ext_MeanEMG_Amplitude)	.505	.348	.529	1.890

a. Dependent Variable: FollowUp_Extension_Torque

Excluded Variables[a]

Model		Beta In	t	Sig.	Partial Correlation	Collinearity .
						Tolerance
1	SMEAN (Ext_MeanEMG_Amplitude)	.478[b]	2.269	.038	.505	.529

Excluded Variables[a]

Model		Collinearity Statistics	
		VIF	Minimum Tolerance
1	SMEAN (Ext_MeanEMG_Amplitude)	1.890	.390

a. Dependent Variable: FollowUp_Extension_Torque

b. Predictors in the Model: (Constant), Weight_kg, Age_years, Gender

Collinearity Diagnostics[a]

Model	Dimension	Eigenvalue	Condition Index	Variance Proportions			
				(Constant)	Gender	Age_years	Weight_kg
1	1	3.440	1.000	.00	.02	.00	.00
	2	.528	2.552	.00	.57	.00	.00
	3	.023	12.187	.30	.00	.85	.02
	4	.009	19.861	.70	.41	.14	.97
2	1	4.323	1.000	.00	.01	.00	.00
	2	.529	2.858	.00	.40	.00	.00
	3	.127	5.832	.00	.03	.04	.01
	4	.014	17.529	.06	.01	.91	.33
	5	.006	26.561	.94	.55	.05	.67

Collinearity Diagnostics[a]

Model	Dimension	Variance ...
		SMEAN (Ext_MeanEMG _Amplitude)
1	1	
	2	
	3	
	4	
2	1	.00
	2	.00
	3	.38
	4	.21
	5	.41

a. Dependent Variable: FollowUp_Extension_Torque

Residuals Statistics[a]

	Minimum	Maximum	Mean	Std. Deviation	N
Predicted Value	57.4008	168.3841	108.8459	33.09996	20
Std. Predicted Value	-1.554	1.799	.000	1.000	20
Standard Error of Predicted Value	8.284	19.143	13.415	3.017	20
Adjusted Predicted Value	45.7477	177.3672	107.9861	33.44067	20
Residual	-37.34449	49.99912	.00000	24.40580	20
Std. Residual	-1.360	1.820	.000	.889	20
Stud. Residual	-1.437	2.402	.014	1.043	20
Deleted Residual	-41.74716	87.04694	.85981	34.18938	20
Stud. Deleted Residual	-1.496	2.958	.039	1.124	20
Mahal. Distance	.778	8.278	3.800	2.073	20
Cook's Distance	.003	.855	.088	.187	20
Centered Leverage Value	.041	.436	.200	.109	20

a. Dependent Variable: FollowUp_Extension_Torque

Charts

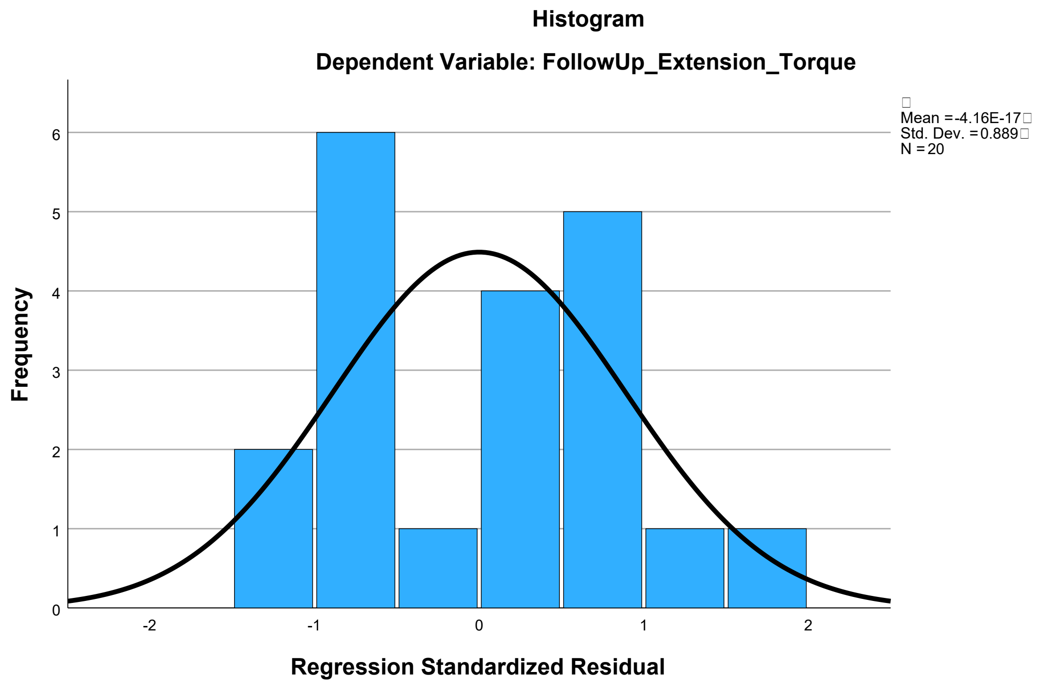

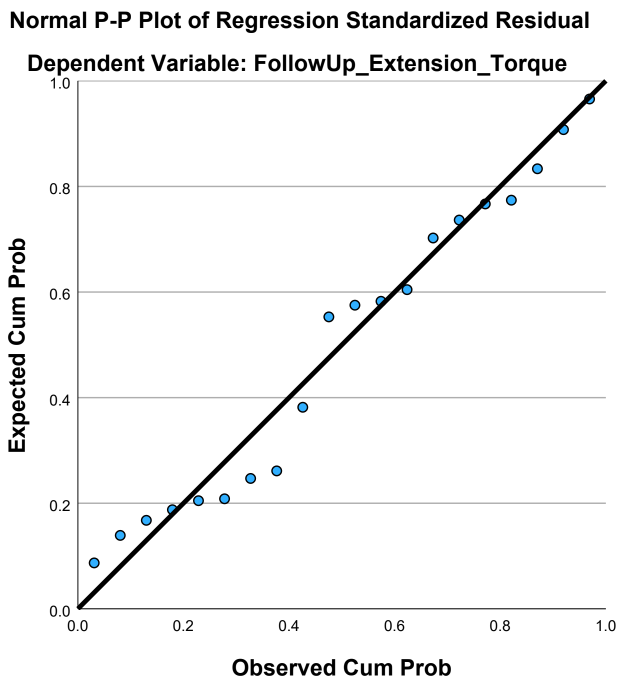
Normal P-P Plot of Regression Standardized Residual
Dependent Variable: FollowUp_Extension_Torque
Expected Cum Prob
Observed Cum Prob
0.0
0.2
0.4
0.6
0.8
1.0

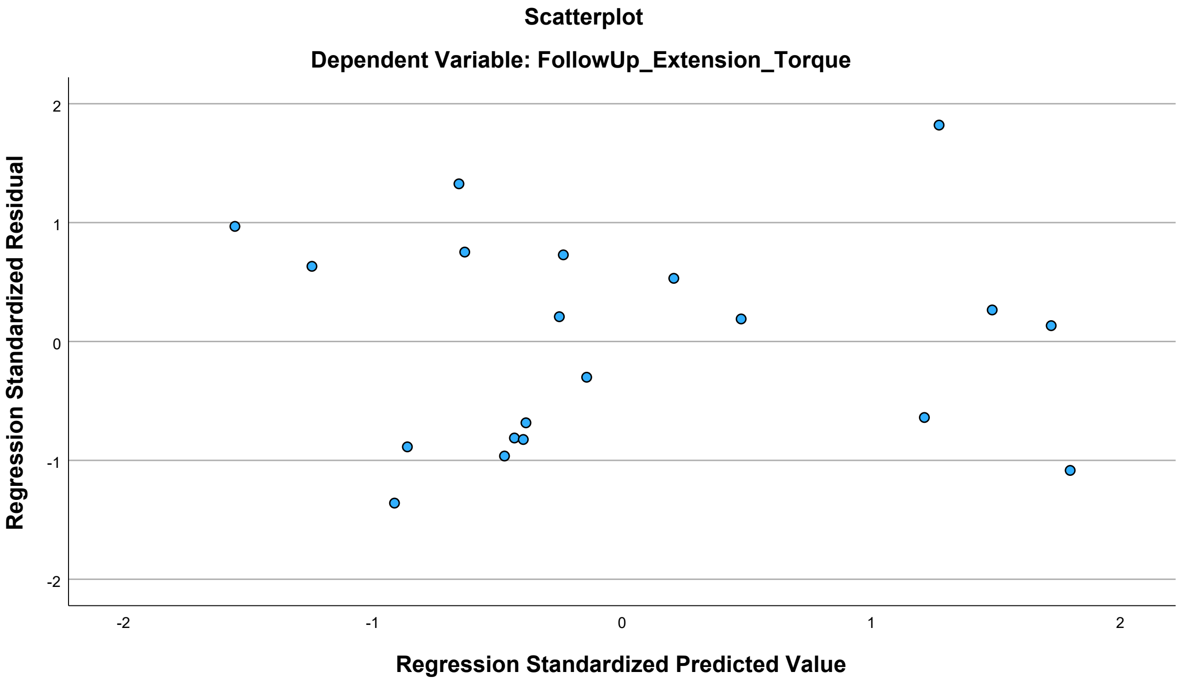
Scatterplot
Dependent Variable: FollowUp_Extension_Torque
Regression Standardized Residual
Regression Standardized Predicted Value
-2
-1
0
1
2

Regression

Descriptive Statistics

	Mean	Std. Deviation	N
FollowUp_IDFlexion_NoOutliers	99.9665	33.60159	20
Age_years	33.2500	7.35473	20
Gender	1.3500	.48936	20
Weight_kg	70.8000	12.72213	20
SMEAN(Coactivation_Flex)	180.5984	158.72698	20
Baseline_Flexion_Nm	86.2770	42.66316	20
Baseline_Pre_Sf36_Avrg_physical	62.7500	16.97328	20
Baseline_pain_pastWeek_NPRS	4.5500	2.16370	20

Correlations

		FollowUp_IDFlexion_NoOutliers	Age_years	Gender	Weight_kg
Pearson Correlation	FollowUp_IDFlexion_NoOutliers	1.000	.200	.755	.517
	Age_years	.200	1.000	.165	.493
	Gender	.755	.165	1.000	.637
	Weight_kg	.517	.493	.637	1.000
	SMEAN(Coactivation_Flex)	-.510	.337	-.271	.040
	Baseline_Flexion_Nm	.832	-.001	.626	.492
	Baseline_Pre_Sf36_Avrg_physical	.430	-.143	.227	.140
	Baseline_pain_pastWeek_NPRS	-.412	-.026	-.191	-.068
Sig. (1-tailed)	FollowUp_IDFlexion_NoOutliers	.	.199	<.001	.010
	Age_years	.199	.	.244	.014
	Gender	.000	.244	.	.001
	Weight_kg	.010	.014	.001	.
	SMEAN(Coactivation_Flex)	.011	.073	.123	.433
	Baseline_Flexion_Nm	.000	.498	.002	.014
	Baseline_Pre_Sf36_Avrg_physical	.029	.274	.168	.277
	Baseline_pain_pastWeek_NPRS	.035	.457	.209	.387
N	FollowUp_IDFlexion_NoOutliers	20	20	20	20
	Age_years	20	20	20	20
	Gender	20	20	20	20
	Weight_kg	20	20	20	20
	SMEAN(Coactivation_Flex)	20	20	20	20

Correlations

		SMEAN (Coactivation_Flex)	Baseline_Flexion_Nm	Baseline_Pre_Sf36_Avrg_physical
Pearson Correlation	FollowUp_IDFlexion_NoOutliers	-.510	.832	.430
	Age_years	.337	-.001	-.143
	Gender	-.271	.626	.227
	Weight_kg	.040	.492	.140
	SMEAN(Coactivation_Flex)	1.000	-.584	-.235
	Baseline_Flexion_Nm	-.584	1.000	.220
	Baseline_Pre_Sf36_Avrg_physical	-.235	.220	1.000
	Baseline_pain_pastWeek_NPRS	-.076	-.361	.050
Sig. (1-tailed)	FollowUp_IDFlexion_NoOutliers	.011	<.001	.029
	Age_years	.073	.498	.274
	Gender	.123	.002	.168
	Weight_kg	.433	.014	.277
	SMEAN(Coactivation_Flex)	.	.003	.159
	Baseline_Flexion_Nm	.003	.	.176
	Baseline_Pre_Sf36_Avrg_physical	.159	.176	.
	Baseline_pain_pastWeek_NPRS	.374	.059	.417
N	FollowUp_IDFlexion_NoOutliers	20	20	20
	Age_years	20	20	20
	Gender	20	20	20
	Weight_kg	20	20	20
	SMEAN(Coactivation_Flex)	20	20	20

Correlations

		Baseline_pain_pastWeek_NPRS
Pearson Correlation	FollowUp_IDFlexion_NoOutliers	-.412
	Age_years	-.026
	Gender	-.191
	Weight_kg	-.068
	SMEAN(Coactivation_Flex)	-.076
	Baseline_Flexion_Nm	-.361
	Baseline_Pre_Sf36_Avrg_physical	.050
	Baseline_pain_pastWeek_NPRS	1.000
Sig. (1-tailed)	FollowUp_IDFlexion_NoOutliers	.035
	Age_years	.457
	Gender	.209
	Weight_kg	.387
	SMEAN(Coactivation_Flex)	.374
	Baseline_Flexion_Nm	.059
	Baseline_Pre_Sf36_Avrg_physical	.417
	Baseline_pain_pastWeek_NPRS	.
N	FollowUp_IDFlexion_NoOutliers	20
	Age_years	20
	Gender	20
	Weight_kg	20
	SMEAN(Coactivation_Flex)	20

Correlations

		FollowUp_IDFlexion_NoOutliers	Age_years	Gender	Weight_kg
	Baseline_Flexion_Nm	20	20	20	20
	Baseline_Pre_Sf36_Avrg_physical	20	20	20	20
	Baseline_pain_pastWeek_NPRS	20	20	20	20

Correlations

		SMEAN (Coactivation_Flex)	Baseline_Flexion_Nm	Baseline_Pre_Sf36_Avrg_physical
	Baseline_Flexion_Nm	20	20	20
	Baseline_Pre_Sf36_Avrg_physical	20	20	20
	Baseline_pain_pastWeek_NPRS	20	20	20

Correlations

		Baseline_pain_pastWeek_NPRS
	Baseline_Flexion_Nm	20
	Baseline_Pre_Sf36_Avrg_physical	20
	Baseline_pain_pastWeek_NPRS	20

Variables Entered/Removed[a]

Model	Variables Entered	Variables Removed	Method
1	Weight_kg, Age_years, Gender[b]	.	Enter
2	SMEAN (Coactivation_Flex)[b]	.	Enter
3	Baseline_Flexion_Nm[b]	.	Enter
4	Baseline_Pre_Sf36_Avrg_physical[b]	.	Enter
5	Baseline_pain_pastWeek_NPRS[b]	.	Enter

a. Dependent Variable: FollowUp_IDFlexion_NoOutliers

b. All requested variables entered.

Model Summary[f]

Model	R	R Square	Adjusted R Square	Std. Error of the Estimate	Change Statistics: R Square Change	F Change	df1	df2	Sig. F Change	Durbin-Watson
1	.759[a]	.576	.496	23.85053	.576	7.237	3	16	.003	
2	.850[b]	.722	.648	19.92277	.147	7.931	1	15	.013	
3	.910[c]	.829	.767	16.20625	.106	8.669	1	14	.011	
4	.943[d]	.890	.839	13.47636	.061	7.246	1	13	.018	
5	.966[e]	.933	.893	10.98388	.043	7.569	1	12	.018	2.747

a. Predictors: (Constant), Weight_kg, Age_years, Gender

b. Predictors: (Constant), Weight_kg, Age_years, Gender, SMEAN(Coactivation_Flex)

c. Predictors: (Constant), Weight_kg, Age_years, Gender, SMEAN(Coactivation_Flex), Baseline_Flexion_Nm

d. Predictors: (Constant), Weight_kg, Age_years, Gender, SMEAN(Coactivation_Flex), Baseline_Flexion_Nm, Baseline_Pre_Sf

e. Predictors: (Constant), Weight_kg, Age_years, Gender, SMEAN(Coactivation_Flex), Baseline_Flexion_Nm, Baseline_Pre_Sf
Baseline_pain_pastWeek_NPRS

f. Dependent Variable: FollowUp_IDFlexion_NoOutliers

ANOVA[a]

Model		Sum of Squares	df	Mean Square	F	Sig.
1	Regression	12350.699	3	4116.900	7.237	.003[b]
	Residual	9101.568	16	568.848		
	Total	21452.267	19			
2	Regression	15498.515	4	3874.629	9.762	<.001[c]
	Residual	5953.752	15	396.917		
	Total	21452.267	19			
3	Regression	17775.273	5	3555.055	13.536	<.001[d]
	Residual	3676.994	14	262.642		
	Total	21452.267	19			
4	Regression	19091.308	6	3181.885	17.520	<.001[e]
	Residual	2360.959	13	181.612		
	Total	21452.267	19			
5	Regression	20004.519	7	2857.788	23.687	<.001[f]
	Residual	1447.748	12	120.646		
	Total	21452.267	19			

a. Dependent Variable: FollowUp_IDFlexion_NoOutliers

b. Predictors: (Constant), Weight_kg, Age_years, Gender

c. Predictors: (Constant), Weight_kg, Age_years, Gender, SMEAN(Coactivation_Flex)

d. Predictors: (Constant), Weight_kg, Age_years, Gender, SMEAN(Coactivation_Flex), Baseline_Flexion_Nm

e. Predictors: (Constant), Weight_kg, Age_years, Gender, SMEAN(Coactivation_Flex), Baseline_Flexion_Nm, Baseline_Pre_Sf36_Avrg_physical

f. Predictors: (Constant), Weight_kg, Age_years, Gender, SMEAN(Coactivation_Flex), Baseline_Flexion_Nm, Baseline_Pre_Sf36_Avrg_physical, Baseline_pain_pastWeek_NPRS

Coefficients[a]

Model		Unstandardized Coefficients B	Unstandardized Coefficients Std. Error	Standardized Coefficients Beta	t
1	(Constant)	18.380	33.029		.556
	Age_years	.334	.877	.073	.381
	Gender	50.445	14.887	.735	3.389
	Weight_kg	.034	.649	.013	.052
2	(Constant)	18.181	27.590		.659
	Age_years	1.006	.771	.220	1.306
	Gender	37.966	13.201	.553	2.876
	Weight_kg	.195	.545	.074	.357
	SMEAN(Coactivation_Flex)	-.093	.033	-.437	-2.816
3	(Constant)	17.148	22.446		.764
	Age_years	1.148	.629	.251	1.826
	Gender	28.326	11.227	.413	2.523
	Weight_kg	-.347	.480	-.132	-.724
	SMEAN(Coactivation_Flex)	-.034	.033	-.159	-1.007
	Baseline_Flexion_Nm	.430	.146	.546	2.944
4	(Constant)	-13.673	21.897		-.624
	Age_years	1.389	.530	.304	2.618
	Gender	26.126	9.371	.380	2.788
	Weight_kg	-.483	.402	-.183	-1.200
	SMEAN(Coactivation_Flex)	-.024	.028	-.113	-.854
	Baseline_Flexion_Nm	.441	.121	.560	3.632
	Baseline_Pre_Sf36_Avrg_physical	.521	.193	.263	2.692
5	(Constant)	5.624	19.176		.293
	Age_years	1.364	.432	.298	3.154
	Gender	24.261	7.668	.353	3.164
	Weight_kg	-.157	.349	-.059	-.450
	SMEAN(Coactivation_Flex)	-.059	.026	-.279	-2.256
	Baseline_Flexion_Nm	.254	.120	.323	2.118
	Baseline_Pre_Sf36_Avrg_physical	.549	.158	.277	3.473
	Baseline_pain_pastWeek_NPRS	-4.034	1.466	-.260	-2.751

Coefficients[a]

Model		Sig.	95.0% Confidence Interval for B Lower Bound	Upper Bound	Correlations Zero-order
1	(Constant)	.586	-51.638	88.399	
	Age_years	.708	-1.525	2.193	.200
	Gender	.004	18.887	82.004	.755
	Weight_kg	.959	-1.342	1.410	.517
2	(Constant)	.520	-40.625	76.988	
	Age_years	.211	-.636	2.649	.200
	Gender	.012	9.829	66.104	.755
	Weight_kg	.726	-.967	1.357	.517
	SMEAN(Coactivation_Flex)	.013	-.163	-.022	-.510
3	(Constant)	.458	-30.993	65.290	
	Age_years	.089	-.200	2.496	.200
	Gender	.024	4.247	52.404	.755
	Weight_kg	.481	-1.377	.682	.517
	SMEAN(Coactivation_Flex)	.331	-.105	.038	-.510
	Baseline_Flexion_Nm	.011	.117	.743	.832
4	(Constant)	.543	-60.978	33.632	
	Age_years	.021	.243	2.534	.200
	Gender	.015	5.881	46.371	.755
	Weight_kg	.251	-1.353	.386	.517
	SMEAN(Coactivation_Flex)	.408	-.084	.037	-.510
	Baseline_Flexion_Nm	.003	.179	.704	.832
	Baseline_Pre_Sf36_Avrg_physical	.018	.103	.938	.430
5	(Constant)	.774	-36.156	47.405	
	Age_years	.008	.422	2.306	.200
	Gender	.008	7.554	40.968	.755
	Weight_kg	.661	-.917	.603	.517
	SMEAN(Coactivation_Flex)	.044	-.116	-.002	-.510
	Baseline_Flexion_Nm	.056	-.007	.516	.832
	Baseline_Pre_Sf36_Avrg_physical	.005	.204	.893	.430
	Baseline_pain_pastWeek_NPRS	.018	-7.229	-.839	-.412

Coefficients[a]

Model		Correlations		Collinearity Statistics	
		Partial	Part	Tolerance	VIF
1	(Constant)				
	Age_years	.095	.062	.720	1.390
	Gender	.646	.552	.564	1.773
	Weight_kg	.013	.008	.439	2.278
2	(Constant)				
	Age_years	.320	.178	.650	1.537
	Gender	.596	.391	.501	1.998
	Weight_kg	.092	.049	.434	2.303
	SMEAN(Coactivation_Flex)	-.588	-.383	.768	1.302
3	(Constant)				
	Age_years	.439	.202	.647	1.546
	Gender	.559	.279	.458	2.183
	Weight_kg	-.190	-.080	.370	2.700
	SMEAN(Coactivation_Flex)	-.260	-.111	.492	2.032
	Baseline_Flexion_Nm	.618	.326	.356	2.806
4	(Constant)				
	Age_years	.588	.241	.628	1.592
	Gender	.612	.257	.455	2.200
	Weight_kg	-.316	-.110	.365	2.743
	SMEAN(Coactivation_Flex)	-.231	-.079	.484	2.066
	Baseline_Flexion_Nm	.710	.334	.356	2.810
	Baseline_Pre_Sf36_Avrg_physical	.598	.248	.887	1.127
5	(Constant)				
	Age_years	.673	.237	.628	1.592
	Gender	.674	.237	.451	2.217
	Weight_kg	-.129	-.034	.322	3.101
	SMEAN(Coactivation_Flex)	-.546	-.169	.369	2.710
	Baseline_Flexion_Nm	.522	.159	.242	4.132
	Baseline_Pre_Sf36_Avrg_physical	.708	.260	.884	1.132
	Baseline_pain_pastWeek_NPRS	-.622	-.206	.631	1.585

a. Dependent Variable: FollowUp_IDFlexion_NoOutliers

Excluded Variables[a]

Model		Beta In	t	Sig.	Partial Correlation	Collinearity . Tolerance
1	SMEAN(Coactivation_Flex)	-.437[b]	-2.816	.013	-.588	.768
	Baseline_Flexion_Nm	.658[b]	4.430	<.001	.753	.556
	Baseline_Pre_Sf36_Avrg_physical	.300[b]	1.891	.078	.439	.905
	Baseline_pain_pastWeek_NPRS	-.281[b]	-1.804	.091	-.422	.957
2	Baseline_Flexion_Nm	.546[c]	2.944	.011	.618	.356
	Baseline_Pre_Sf36_Avrg_physical	.251[c]	1.878	.081	.449	.888
	Baseline_pain_pastWeek_NPRS	-.353[c]	-3.178	.007	-.647	.933
3	Baseline_Pre_Sf36_Avrg_physical	.263[d]	2.692	.018	.598	.887
	Baseline_pain_pastWeek_NPRS	-.239[d]	-1.861	.085	-.459	.633
4	Baseline_pain_pastWeek_NPRS	-.260[e]	-2.751	.018	-.622	.631

Excluded Variables[a]

Model		Collinearity Statistics VIF	Minimum Tolerance
1	SMEAN(Coactivation_Flex)	1.302	.434
	Baseline_Flexion_Nm	1.798	.409
	Baseline_Pre_Sf36_Avrg_physical	1.105	.434
	Baseline_pain_pastWeek_NPRS	1.045	.436
2	Baseline_Flexion_Nm	2.806	.356
	Baseline_Pre_Sf36_Avrg_physical	1.126	.428
	Baseline_pain_pastWeek_NPRS	1.072	.430
3	Baseline_Pre_Sf36_Avrg_physical	1.127	.356
	Baseline_pain_pastWeek_NPRS	1.579	.242
4	Baseline_pain_pastWeek_NPRS	1.585	.242

a. Dependent Variable: FollowUp_IDFlexion_NoOutliers

b. Predictors in the Model: (Constant), Weight_kg, Age_years, Gender

c. Predictors in the Model: (Constant), Weight_kg, Age_years, Gender, SMEAN(Coactivation_Flex)

d. Predictors in the Model: (Constant), Weight_kg, Age_years, Gender, SMEAN(Coactivation_Flex), Baseline_Flexion_Nm

e. Predictors in the Model: (Constant), Weight_kg, Age_years, Gender, SMEAN(Coactivation_Flex), Baseline_Flexion_Nm, Baseline_Pre_Sf36_Avrg_physical

Collinearity Diagnostics[a]

				Variance Proportions			
Model	Dimension	Eigenvalue	Condition Index	(Constant)	Age_years	Gender	Weight_kg
1	1	3.893	1.000	.00	.00	.00	.00
	2	.076	7.173	.03	.09	.57	.00
	3	.022	13.323	.62	.68	.03	.00
	4	.010	20.031	.35	.23	.40	1.00
2	1	4.516	1.000	.00	.00	.00	.00
	2	.398	3.367	.00	.00	.02	.00
	3	.055	9.053	.08	.10	.58	.00
	4	.021	14.562	.56	.72	.01	.00
	5	.010	21.627	.35	.18	.39	.99
3	1	5.334	1.000	.00	.00	.00	.00
	2	.533	3.163	.00	.00	.01	.00
	3	.059	9.505	.10	.12	.18	.00
	4	.044	11.000	.00	.00	.61	.00
	5	.021	15.890	.60	.68	.00	.00
	6	.009	24.684	.29	.19	.21	.99
4	1	6.262	1.000	.00	.00	.00	.00
	2	.538	3.411	.00	.00	.00	.00
	3	.085	8.583	.02	.00	.08	.00
	4	.048	11.441	.03	.23	.32	.00
	5	.043	12.038	.00	.07	.39	.00
	6	.015	20.279	.75	.51	.00	.01
	7	.009	26.746	.21	.19	.20	.98
5	1	7.064	1.000	.00	.00	.00	.00
	2	.539	3.619	.00	.00	.00	.00
	3	.233	5.502	.00	.00	.01	.00
	4	.064	10.515	.00	.02	.07	.00
	5	.047	12.315	.02	.10	.66	.00
	6	.031	15.024	.00	.41	.04	.00
	7	.013	23.043	.95	.23	.02	.00
	8	.008	29.737	.02	.24	.19	.99

Collinearity Diagnostics[a]

Model	Dimension	Variance Proportions SMEAN (Coactivation_Flex)	Baseline_Flexion_Nm	Baseline_Pre_Sf36_Avrg_physical	Baseline_pain_pastWeek_NPRS
1	1				
	2				
	3				
	4				
2	1	.01			
	2	.65			
	3	.30			
	4	.04			
	5	.01			
3	1	.00	.00		
	2	.28	.03		
	3	.50	.18		
	4	.14	.66		
	5	.01	.01		
	6	.07	.11		
4	1	.00	.00	.00	
	2	.28	.03	.00	
	3	.15	.16	.36	
	4	.17	.01	.20	
	5	.32	.67	.06	
	6	.01	.02	.37	
	7	.07	.12	.00	
5	1	.00	.00	.00	.00
	2	.21	.02	.00	.00
	3	.06	.02	.00	.34
	4	.00	.01	.68	.11
	5	.01	.12	.03	.01
	6	.48	.48	.01	.29
	7	.06	.09	.27	.14
	8	.18	.26	.01	.12

a. Dependent Variable: FollowUp_IDFlexion_NoOutliers

Residuals Statistics[a]

	Minimum	Maximum	Mean	Std. Deviation	N
Predicted Value	42.3045	162.7410	99.9665	32.44795	20
Std. Predicted Value	-1.777	1.935	.000	1.000	20
Standard Error of Predicted Value	4.643	10.048	6.815	1.384	20
Adjusted Predicted Value	28.4706	169.0970	99.2243	33.91850	20
Residual	-15.02232	15.58423	.00000	8.72910	20
Std. Residual	-1.368	1.419	.000	.795	20
Stud. Residual	-1.667	1.734	.020	1.003	20
Deleted Residual	-22.31172	23.90325	.74216	14.38128	20
Stud. Deleted Residual	-1.820	1.917	.025	1.053	20
Mahal. Distance	2.445	14.952	6.650	3.132	20
Cook's Distance	.000	.237	.085	.079	20
Centered Leverage Value	.129	.787	.350	.165	20

a. Dependent Variable: FollowUp_IDFlexion_NoOutliers

Charts

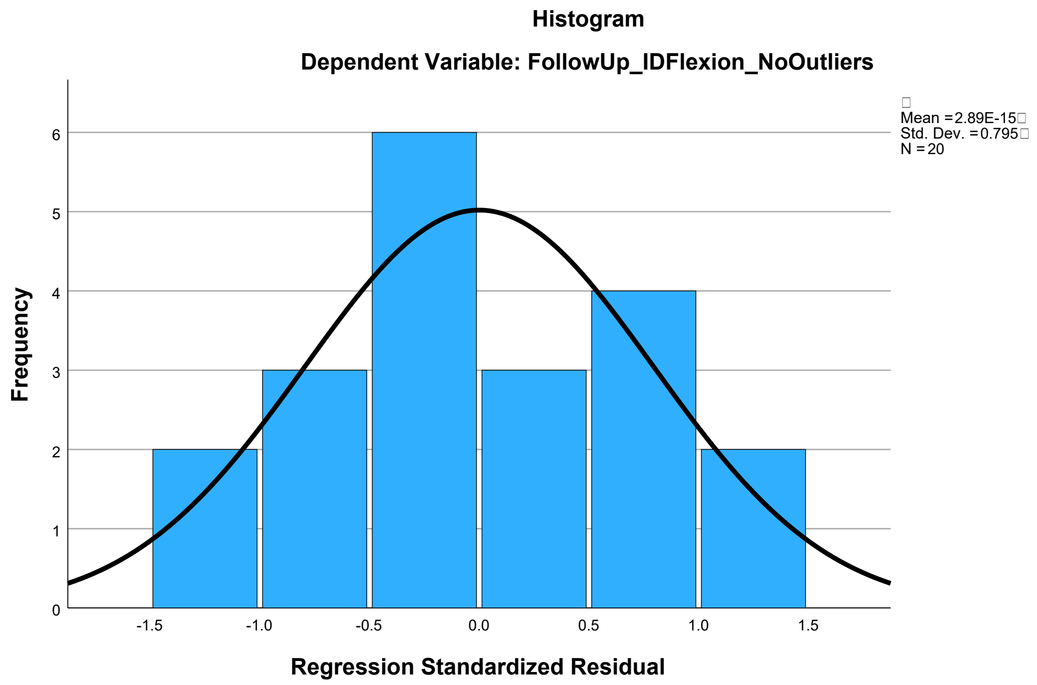

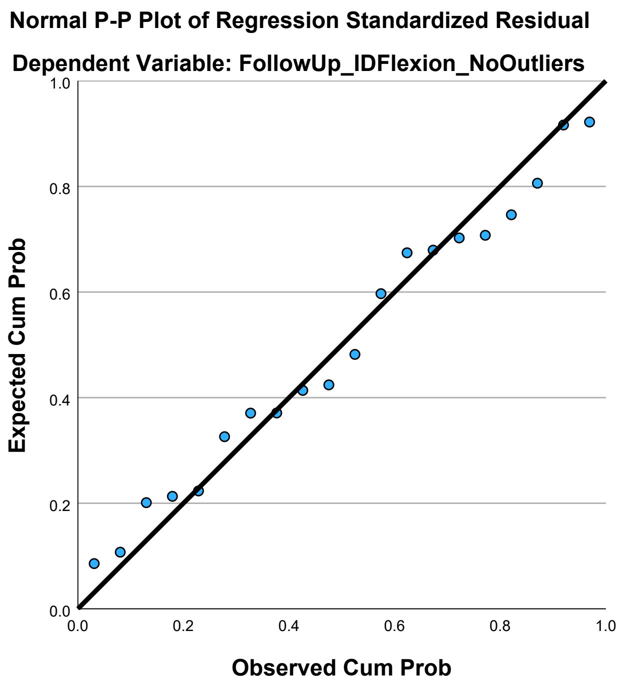
Normal P-P Plot of Regression Standardized Residual
Dependent Variable: FollowUp_IDFlexion_NoOutliers
Expected Cum Prob
Observed Cum Prob
0.0
0.2
0.4
0.6
0.8
1.0

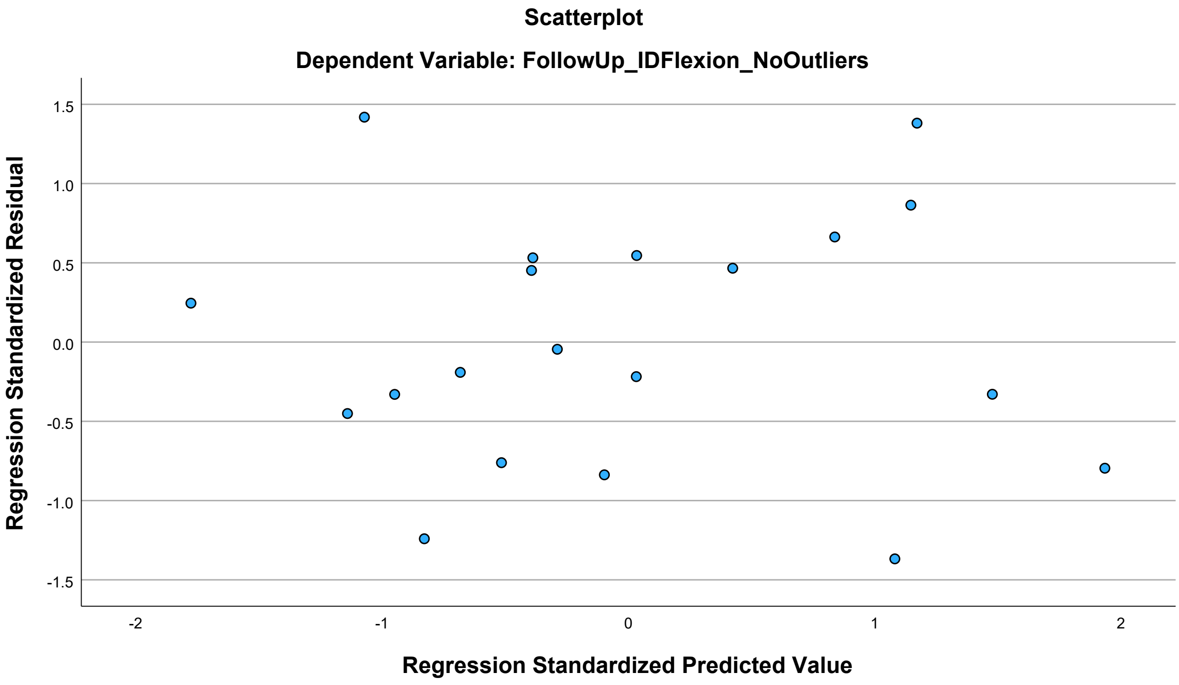
Scatterplot
Dependent Variable: FollowUp_IDFlexion_NoOutliers
Regression Standardized Residual
Regression Standardized Predicted Value
1.5
1.0
0.5
0.0
-0.5
-1.0
-1.5
-2
-1
0
1
2

Supplementary material 4: STROBE Statement—checklist of items that should be included in reports of observational studies

	Item No.	Recommendation	Page No.
**Title and abstract**	1	(*a*) Indicate the study's design with a commonly used term in the title or the abstract	2
		(*b*) Provide in the abstract an informative and balanced summary of what was done and what was found	2
**Introduction**			
Background/rationale	2	Explain the scientific background and rationale for the investigation being reported	3-5
Objectives	3	State specific objectives, including any prespecified hypotheses	5
**Methods**			
Study design	4	Present key elements of study design early in the paper	21
Setting	5	Describe the setting, locations, and relevant dates, including periods of recruitment, exposure, follow-up, and data collection	21
Participants	6	(*a*) *Cohort study*—Give the eligibility criteria, and the sources and methods of selection of participants. Describe methods of follow-up<br>*Case-control study*—Give the eligibility criteria, and the sources and methods of case ascertainment and control selection. Give the rationale for the choice of cases and controls<br>*Cross-sectional study*—Give the eligibility criteria, and the sources and methods of selection of participants	21-22
		(*b*) *Cohort study*—For matched studies, give matching criteria and number of exposed and unexposed<br>*Case-control study*—For matched studies, give matching criteria and the number of controls per case	
Variables	7	Clearly define all outcomes, exposures, predictors, potential confounders, and effect modifiers. Give diagnostic criteria, if applicable	22-26
Data sources/ measurement	8*	For each variable of interest, give sources of data and details of methods of assessment (measurement). Describe comparability of assessment methods if there is more than one group	22-26
Bias	9	Describe any efforts to address potential sources of bias	19
Study size	10	Explain how the study size was arrived at	NA

Continued on next page

Quantitative variables	11	Explain how quantitative variables were handled in the analyses. If applicable, describe which groupings were chosen and why	28
Statistical methods	12	(*a*) Describe all statistical methods, including those used to control for confounding	27-28
		(*b*) Describe any methods used to examine subgroups and interactions	27-28
		(*c*) Explain how missing data were addressed	NA
		(*d*) *Cohort study*—If applicable, explain how loss to follow-up was addressed<br>*Case-control study*—If applicable, explain how matching of cases and controls was addressed<br>*Cross-sectional study*—If applicable, describe analytical methods taking account of sampling strategy	NA
		(*e*) Describe any sensitivity analyses	NA
**Results**			
Participants	13*	(a) Report numbers of individuals at each stage of study—eg numbers potentially eligible, examined for eligibility, confirmed eligible, included in the study, completing follow-up, and analysed	6
		(b) Give reasons for non-participation at each stage	NA
		(c) Consider use of a flow diagram	
Descriptive data	14*	(a) Give characteristics of study participants (eg demographic, clinical, social) and information on exposures and potential confounders	6-7
		(b) Indicate number of participants with missing data for each variable of interest	NA
		(c) *Cohort study*—Summarise follow-up time (eg, average and total amount)	
Outcome data	15*	*Cohort study*—Report numbers of outcome events or summary measures over time	6-7
		Case-control study—Report numbers in each exposure category, or summary measures of exposure	
		Cross-sectional study—Report numbers of outcome events or summary measures	
Main results	16	(*a*) Give unadjusted estimates and, if applicable, confounder-adjusted estimates and their precision (eg, 95% confidence interval). Make clear which confounders were adjusted for and why they were included	7-13
		(*b*) Report category boundaries when continuous variables were categorized	NA
		(*c*) If relevant, consider translating estimates of relative risk into absolute risk for a meaningful time period	NA

Continued on next page

Other analyses	17	Report other analyses done—eg analyses of subgroups and interactions, and sensitivity analyses	
**Discussion**			
Key results	18	Summarise key results with reference to study objectives	13-17
Limitations	19	Discuss limitations of the study, taking into account sources of potential bias or imprecision. Discuss both direction and magnitude of any potential bias	18-20
Interpretation	20	Give a cautious overall interpretation of results considering objectives, limitations, multiplicity of analyses, results from similar studies, and other relevant evidence	17-18
Generalisability	21	Discuss the generalisability (external validity) of the study results	18-20
**Other information**			
Funding	22	Give the source of funding and the role of the funders for the present study and, if applicable, for the original study on which the present article is based	35

*Give information separately for cases and controls in case-control studies and, if applicable, for exposed and unexposed groups in cohort and cross-sectional studies.

Note: An Explanation and Elaboration article discusses each checklist item and gives methodological background and published examples of transparent reporting. The STROBE checklist is best used in conjunction with this article (freely available on the Web sites of PLoS Medicine at http://www.plosmedicine.org/, Annals of Internal Medicine at http://www.annals.org/, and Epidemiology at http://www.epidem.com/). Information on the STROBE Initiative is available at www.strobe-statement.org.

Supplementary material: 5

1. Initialize:

- Set file names for endurance and MVC data:
  - `filenameEND = '...Endurance.otb+'`
  - `filenameMVC_Flex = '...FlexMVC.otb+'`
  - `filenameMVC_Ext = '...ExtMVC.otb+'`

2. Process MVC Data:

- Call function to get MVC values for extension:

```matlab
[mvcRMS_ext, mvcMNF_ext, pks_ext] = getMVCvalues(filenameMVC_Ext);
```

- Call function to get MVC values for flexion:

```matlab
[mvcRMS_flex, mvcMNF_flex, pks_flex] = getMVCvalues(filenameMVC_Flex);
```

3. Set Parameters:

- Define constants:
  - NPERENDURANCE = 10 (Number of epochs)
  - NCH = 140 (Total number of channels)
  - GAIN = 150
  - BIPOLAR = 2 (Number of bipolar channels)
  - fsamp = 2048 (Sampling frequency)

4. Load and Preprocess Endurance Data:

- Uncompress the endurance file into a temporary directory.
- Read signal data into `XX` matrix.
- Apply gain factor to convert signals to microvolts:

5. Filter Signals:

- Extract bipolar EMG channels:

```matlab
BiEMG = XX(1:2, :);
```

- Design a Butterworth bandpass filter between 10 Hz and 350 Hz.

```matlab
[bemg, aemg] = butter(3, [10, 350] / (fsamp / 2));
BiEMG_filt(1, :) = filtfilt(bemg, aemg, (BiEMG(1, :)));
```

6. Define Electrode Grids:

- Create cell arrays `Col_Matr1` and `Col_Matr2` for electrode indices, adjusted by `BIPOLAR`.

7. Clean and Filter HD-EMG Signals:

- For each column in the grids:
  - For each row:
    - Plot the difference between adjacent channels to visualize signals.
    - Filter and store the processed signal in `Matr_1` or `Matr_2`.

8. Select Start and End Points:

- Plot representative signals and a trigger signal.
- Prompt the user to select start and end points using:

```matlab
[X, Y] = ginput(2);
```

9. Divide Endurance Time into Epochs:

- Calculate total endurance time `ENDURANCE` and epoch step `STEP`.
- Generate indices `ind` for epoch boundaries.

10. Prepare Data for Analysis:

- Convert `Matr_1` and `Matr_2` from cell arrays to matrices.

- Remove zero rows and detrend the data.

- Normalize signals with respect to MVC RMS values:

```matlab
M1_norm = M1 ./ mvcRMS_ext(1);
```

11. Calculate Endurance Metrics:

- Extract endurance portions of the signals based on `X`.

- Define window size for RMS calculation:

- Calculate RMS and MNF over the entire endurance period for each signal.

12. Calculate Epoch Metrics:

- For each epoch:

  - Calculate RMS and MNF for the filtered signals.

  - Normalize RMS values by MVC RMS.

13. Organize Results:

- Create tables to store calculated metrics (RMS, MNF) for endurance and epochs.

- Compile MVC results, including RMS, MNF, and peak forces.

14. Save Results:

- Store all results in a structured variable `results`.

- Save the results to a file:

```matlab
save(['Results_', filenameEND, '.mat'], 'results');
```

Supplementary material 6:

Training load progression: baseline and follow-up

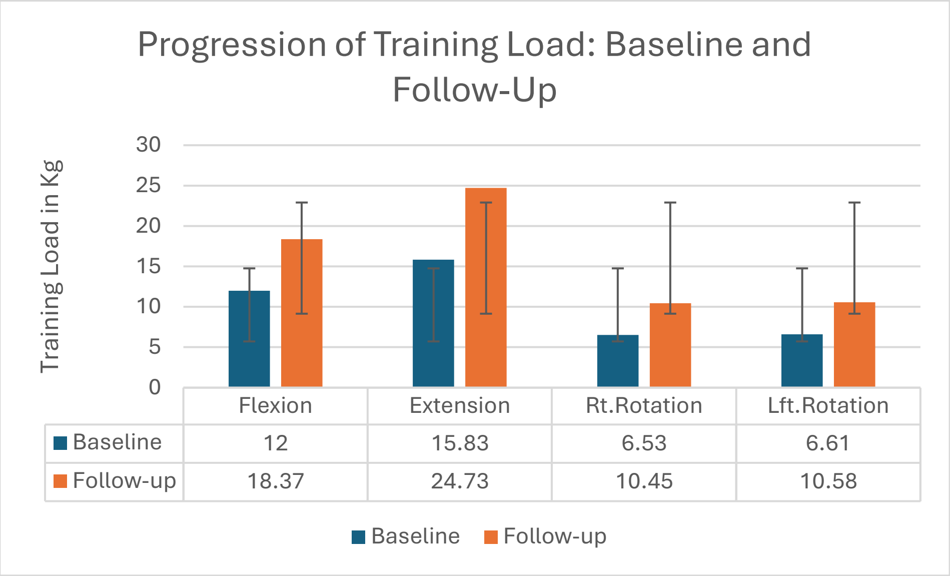

Supplement: Supplementary file 1 — Supplementary Information. [file 41598_2025_93280_MOESM1_ESM.pdf]
